# Complete Genome Sequence and Comparative Analysis of *Synechococcus* sp. CS-601 (SynAce01), a Cold-Adapted Cyanobacterium from an Oligotrophic Antarctic Habitat

**DOI:** 10.3390/ijms20010152

**Published:** 2019-01-03

**Authors:** Jie Tang, Lian-Ming Du, Yuan-Mei Liang, Maurycy Daroch

**Affiliations:** 1 Key Laboratory of Coarse Cereal Processing, Ministry of Agriculture and Rural Affairs, School of Pharmacy and Biological Engineering, Chengdu University, Chengdu 610106, China; tangjie@cdu.edu.cn (J.T.); dulianming@cdu.edu.cn (L.-M.D.); 2 Shenzhen Aone Medical Laboratory Co Ltd, Shenzhen 518107, China; 3 School of Environment and Energy, Peking University Shenzhen Graduate School, Shenzhen 518055, China; lym@sz.pku.edu.cn

**Keywords:** *Synechococcus*, psychrotroph, comparative genomics, cold adaptation, salinity adaptation, oligotrophic environment, Antarctica, high latitudes, cryosphere

## Abstract

Marine picocyanobacteria belonging to Synechococcus are major contributors to the global carbon cycle, however the genomic information of its cold-adapted members has been lacking to date. To fill this void the genome of a cold-adapted planktonic cyanobacterium *Synechococcus* sp. CS-601 (SynAce01) has been sequenced. The genome of the strain contains a single chromosome of approximately 2.75 MBp and GC content of 63.92%. Gene prediction yielded 2984 protein coding sequences and 44 tRNA genes. The genome contained evidence of horizontal gene transfer events during its evolution. CS-601 appears as a transport generalist with some specific adaptation to an oligotrophic marine environment. It has a broad repertoire of transporters of both inorganic and organic nutrients to survive in inhospitable environments. The cold adaptation of the strain exhibited characteristics of a psychrotroph rather than psychrophile. Its salt adaptation strategy is likely to rely on the uptake and synthesis of osmolytes, like glycerol or glycine betaine. Overall, the genome reveals two distinct patterns of adaptation to the inhospitable environment of Antarctica. Adaptation to an oligotrophic marine environment is likely due to an abundance of genes, probably acquired horizontally, that are associated with increased transport of nutrients, osmolytes, and light harvesting. On the other hand, adaptations to low temperatures are likely due to prolonged evolutionary changes.

## 1. Introduction

Marine picocyanobacteria belonging to *Synechococcus* and *Prochlorococcus* are major contributors to the global carbon cycle and may contribute up to half of the total biologically-fixed carbon in certain areas [1,2]. Of the two, the genus *Synechococcus* shows significantly broader geographic distribution ranging from tropical waters to high latitudes of Arctic and Antarctic regions [1,3]. Whilst there is an abundance of studies regarding cyanobacteria of tropical and temperate regions, the studies of extremophilic cyanobacteria are somehow less abundant with the majority of these focusing on hot spring communities and, more recently, isolates [4,5]. Cold-adapted strains of *Synechococcus* are among the least studied strains of cyanobacteria with only a handful of examples available in the literature [6,7,8], despite their being predominately responsible for carbon sequestration and driving the microbial food chain in these environments [8]. This is likely to be caused by several reasons, namely challenging sampling and isolation, inability to maintain axenic cultures, and slow growth rates. To date only a single genome of cold-adapted cyanobacterium has been published sharing some insights into cold-adaptation features of an Arctic filamentous cyanobacterium *Phormidesmis priestleyi* BC1401 [9]. Genomic details regarding abundant planktonic strains of the cryosphere are still lacking [9].

*Synechococcus* is a polyphyletic genus of cyanobacteria which is expected to undergo an extensive revision in its taxonomy in the near future. As such, it needs expanded sequence information, especially on the whole genome level, about the underrepresented members of the genus to guide further phylogenetic and taxonomic studies and eventual reclassification. To date, there has been no genome sequence available for any of the cold-adapted *Synechococcus* strains. This significantly impacts the possibility of carrying out true phylogenomic studies of the cold-adapted cyanobacteria and hinders understanding how these cyanobacteria cope with multiple environmental pressures in the cryosphere [8]. To fill this gap and reveal the underlying evolutionary adaptations to low temperatures on the genomic level, we have decided to sequence the genome of the *Synechococcus* sp. SynAce01-Ace Lake deposited in the Australian National Algae Culture Collection (ANACC) with the accession number CS-601, as a cold-adapted *Synechococcus* strain with arguably the largest amount of data available to date and originating from a well-described ecosystem of saline Antarctic Ace Lake.

The strain CS-601 exhibits growth characteristics of a psychrotolerant strain with the fastest growth rate at ~20 °C, minimum projected growth temperature at −17 °C, and maximal temperature that allowed growth at 29.5 °C [6]. Our laboratory data confirm the optimal and maximal temperatures regarding the growth. The strain is adaptable to both low (2.5 µmol photons PAR m^−2^·s^−1^) and high light conditions (300 µmol photons PAR m^−2^·s^−1^). The strain requires optimal salinities of 20–30 g·kg^−1^ and is unable to grow at salinities lower than 10 and higher than 50 g·kg^−1^ [6].

Over the past decade, the advent of second-generation sequencing technologies has significantly facilitated the capability to perform sequencing and *de novo* assembly of genomes. Among the sequencing platforms, Illumina’s HiSeq platform was the most commonly used for entire genome sequencing, due to high throughput and accuracy [10]. However, this technology is limited by the number of nucleotides that can be sequenced and base-composition bias in genome coverage [11], resulting in more efforts on scaffolding and gap closing for the sake of genome completeness. Recently, increasing attention has been paid to third-generation sequencing technology by Pacific Biosciences (PacBio) [12]. The PacBio sequencing platform can produce significantly longer read length than that of ‘second-generation’ technologies such as the Illumina HiSeq2000, as long as ~23 kb reported previously, and with current average read lengths reaching 2246 kbp [13]. However, one drawback is also applied to the PacBio platform: the raw data it generated is inherently error-prone, with errors up to 17.9% [14]. It is not surprising that an integrated PacBio- and Illumina-based strategy is often employed for complete genome sequencing [15,16,17], the results of which suggest that performance of integrated approaches was tractable. Therefore, a hybrid approach is probably a feasible, robust, and preferable way to determine a bacterial genome.

In the present study, a whole-genome sequence of *Synechococcus* sp. CS-601 (SynAce01) was performed using a combination of PacBio and Illumina technologies. This is the first complete genome sequence of a cold-adapted *Synechococcus* strain. The gene repertoire of *Synechococcus* sp. SynAce01 had been discussed. In addition, acquisition of the complete genome may provide a better understanding of mobile genetic elements on how genome flexibility contributes to adaptation to various ecological niches. The complete genome also provides insights into the genomic features of the *Synechococcus* group, particularly the survival mechanism from a genomic perspective in oligotrophic, cold, and saline conditions.

## 2. Results and Discussion

### 2.1. General Features of Synechococcus sp. CS-601 (SynAce01) Genome

**Figure 1:**
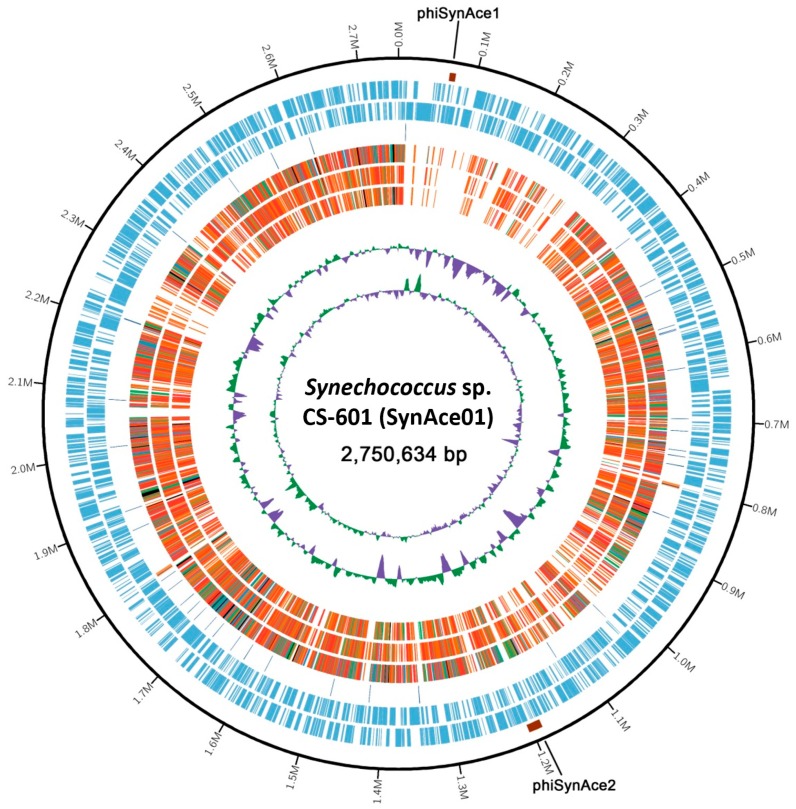
Circular plot of *Synechococcus* sp. CS-601 (SynAce01) genome. Rings are as follows (outer–inner): Two prophages of phiSynAce1 and phiSynAce2; protein-coding sequences (CDS) on plus strand; CDS on minus strand; rRNA (orange) and tRNA (blue); the fifth to seventh circles represent the shared amino acid identities of Basic Local Alignment Sequence Tool-Protein Search (BLASTP) alignments with *Synechococcus* sp. WH8102, *S. elongatus* PCC 7942, and *P. marinus* MED4, respectively; the last two circles represent GC content and GC skew both calculated for a 10-kb window with 1-kb stepping. The colour scheme for the heat map of orthologs is as follows, black, orthologs ≥90% identity; blue, 80–90% identity; green, 70–80% identity; red, 50–70% identity; orange, 30–50% identity.

The complete genome of the CS-601 strain was obtained by a combined assembly of the PacBio and Illumina sequencing system. The genome of the strain (Figure 1) comprises a single circular chromosome with a size of 2,750,634 bp (GC content, 63.92%). Gene prediction and annotation of the strain resulted in 2984 protein-coding sequences (CDS) (Table S1). Functional distribution on gene ontology (GO) categories of these CDS identified in the genome is summarized in Figure S1. Two ribosomal RNA (rrn) operons were detected and 44 tRNA genes were predicted in the SynAce01 chromosome (Table 1). Moreover, the SynAce01 genome, like many prokaryotic genomes, includes many repetitive sequences, e.g., 197 tandem repeats of varying length up to 30 bp. Although fragmented contigs were assembled for these regions using short reads, the longer reads (7 kb on average) produced by the PacBio sequencer correctly reconstructed these repetitive regions, finally providing a comprehensive snapshot of the genome.

### 2.2. Mobile Genetic Elements

Mobile genetic elements played a crucial role in genome evolution, conferring more plasticity to the bacterial genome for adaptation to various environmental conditions. Mobile genetic elements also contributed greatly to horizontal gene transfer (HGT).

In total, 61 ISs (insertion sequences) representing 34 different ISs were identified in the SynAce01 genome. The most frequently observed IS type was the IS3 family (19.67%), followed by the IS1595 family (18.03%) and IS91 family (16.39%). A high content of genes encoding transposase (Table S1) was also observed, indicating that the genetic plasticity of the strain might be determined by intragenomic rearrangements. It was proposed that transpositions play a crucial role in genomic rearrangements and are involved in gene regulation and adaptation processes that determine the directions of microevolutionary processes in cyanobacteria [18].

Two prophage loci were predicted in the chromosome, phiSynAce1 (8.2 kb; positions 65,524–73,790) and phiSynAce2 (18.2 kb; positions 1,186,119–1,204,368). Eight and eleven phage-related genes were identified in these regions, respectively (Table S2). No genes for DNA synthesis were found in the two prophage loci, indicating that these are replication-defective. A 12-bp direct repeat (ACAGGCCAGCGC, positions 1,186,119–1,186,130 and 1,204,368–1,204,379) was found to flank phiSynAce2, which appeared to constitute the core regions of phage attachment (*att*L and *att*R). This result is consistent with conclusion in the previous study that *Synechococcus* may be more subject than *Prochlorococcus* to HGT from phages, as evidenced by the presence of more phage integrases [19].

Clustered, regularly-interspaced short palindromic repeats (CRISPRs) were reported to be a component of many bacterial genomes, and CRISPRs functioned in the interference pathway to preserve genome integrity [20]. In the SynAce01 chromosome, no CRISPRs were detected. The finding was in accord with the existence of prophage-like regions and IS sequences in the genome, since CRISPR interference can limit horizontal gene transfer [21].

In order to detect the HGT events in CS-601 (SynAce01), genes coding for phycocyanin were selected based on our research interest. The aligned and concatenated sequences of *cpcAB* genes of the three *Synechococcus* strains were examined for recombination events using RDP v4.97 software. Recombination events were accepted if at least four different methods detected statistically significant (*p* < 0.05) evidence of recombination. The results showed that two events were detected and CS-601 (SynAce01) was detected as recombinant, with WH8102 as a major parent and PCC7942 as minor parent, and with an unknown as a major parent and PCC7942 as minor parent, respectively. Recombination breakpoints were detected in *cpcA* (position 285) and *cpcB* (position 380). The results indicated that HGT might occur in CS-601 (SynAce01) genome during the evolutionary process.

### 2.3. Gene Contents in Synechococcus sp. CS-601 (SynAce01)

**Figure 2:**
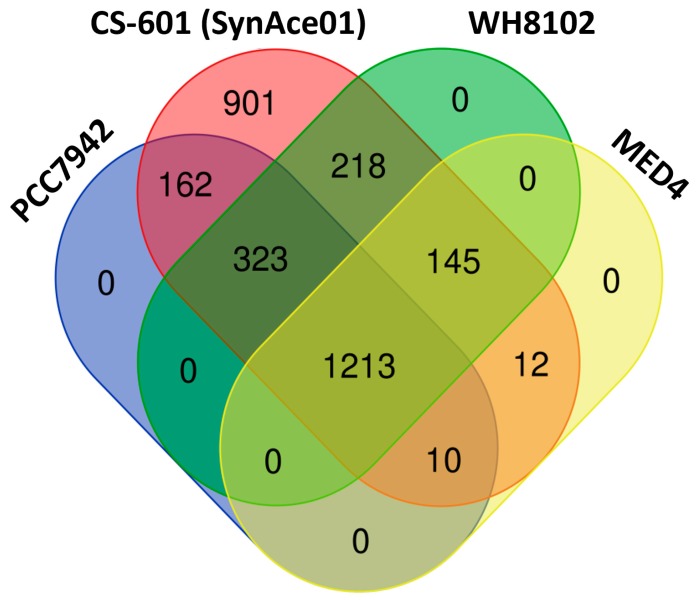
Venn diagram representing the number of orthologous genes among all strains used in this study. *E*-value cut-off of 1E-5 and ≥30% identity was used as thresholds in the all-against-all BLASTP alignments.

**Figure 3:**
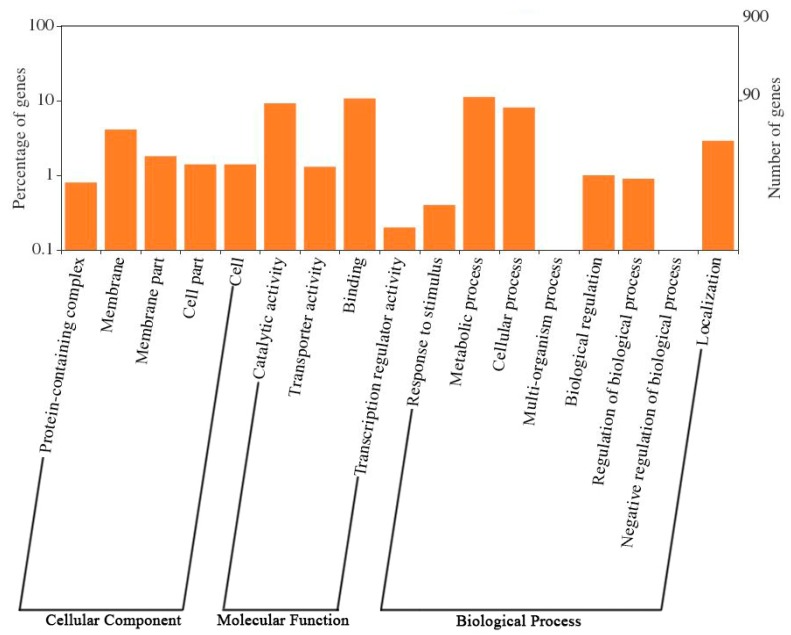
Gene ontology (GO) analysis and functional classification of specific genes in CS-601 (SynAce01) genome identified in this study.

To clarify the gene features of the Antarctic strain and its relatives, a comparative analysis was performed using three strains from genus *Synechococcus* and one strain from genus *Prochlorococcus*. Genome statistics and related information for the four strains are shown in Table 1. An ortholog table (Table S3) was constructed based on all-against-all BLASTP alignment. Figure 2 indicates that 1213 genes were defined to be common to all four genomes, and 901 genes were found only in SynAce01. Further GO analysis of the specific genes showed that these genes were distributed in a wide range of functional categories (Figure 3). The result may suggest the specific ecological strategies of SynAce01 relative to the other three reference strains.

The *Synechococcus* sp. CS-601 (SynAce01) genome indicated the uniqueness of *Synechococcus* swimming motility. None of the proteins (motor or flagella) associated with other forms of prokaryotic motility was found. However, eight ORFs associated with the pilus system of motility proteins were observed, including three twitching motility proteins (homologs of pilT) and pilus assembly proteins (homologs of pilB, *-C*, *-D*, *-Q*, and cpaF). Orthologues of pil are present in WH8102 and PCC7942, but not MED4, while cpaF was only present in SynAce01 and PCC7942. Nevertheless, these ORFs in SynAce01 do not encode the full complement of genes required for pilus assembly and function. Pili components have not been observed in SynAce01 genome.

CS-601 (SynAce01) appeared to be a transporter generalist, with more than one hundred transporter-related genes predicted in the genome (Table S1). Among these transporters, ABC transporters accounted for the majority and a distinct bias was found in P-type ATPase transporter for copper which has only one copy. Functionally, these transporters have been predicted as Na^+^/H^+^, iron, phosphate, amino acid, bicarbonate, CO_2_ transporters, etc.

A number of conserved systems for exporting compounds (e.g., multidrug efflux systems) are found both in the ABC transporter family and the MFS transporter family. CS-601 has a larger number of efflux transporters in the ABC family compared with *Prochlorococcus*. Genes coding an antitoxin system (SA0828 and SA1213) were also found. These results indicated that marine cyanobacteria, despite living in extremely oligotrophic conditions, may still need to export ‘toxins’ produced by other microorganisms. Although some marine *Synechococcus* and *Prochlorococcus* do not seem to have a requirement for zinc detoxification [22], multiple zinc/manganese transporters (e.g., SA2849 and SA2850) were detected in SynAce01 genome.

A two-component regulatory system is an important way for cyanobacteria to sense and respond to the environment, comprising a sensor kinase and a response regulator. Freshwater *Synechocystis* sp. PCC6803 was reported to have 40 pairs of sensor kinase and response regulator [19]. In contrast, CS-601 only has eight histidine kinases and nine response regulators (Table S1). This result is consistent with the genomes of marine WH8102 and MED4, but different from that of freshwater PCC7942 (~30 kinases). Genome analysis of the Antarctic cyanobacterium suggested that SynAce01 may have fewer systems to respond to the changing environment and some sensors may function with more than one regulator, perhaps in light of an economy of regulation from survival aspect.

### 2.4. Cold Adaptation Strategy

Bacteria perceive cold by transmembrane histidine kinases and respond by two-component regulatory systems [23]. Regulating membrane fluidity is a universal strategy to acclimate changing ambient temperature *via* fatty acid profile changes, such as the conversion of saturated fatty acids into unsaturated fatty acids and the preferential synthesis of short-chain, branched-chain, and/or anteiso fatty acids [24,25]. In the CS-601 (SynAce01) genome, eight genes encoding histidine kinases were found, which may act as a multifunctional sensory to control numerous cold-responsive genes as well as responses to osmotic, salt, and oxidative stress [26]. Coupled with histidine kinases, response regulators (such as *rpaA/B*) might be the components of cold perception and transduction system [27]. Another cold-induced regulatory gene found in the strain is the DNA-binding transcriptional regulator, *sfsA* (SA1722), which is involved in regulation of sugar catabolism. However, its exact function in cyanobacteria is unknown [26].

Eight genes (homologs of *desA*, *desC*, *FAD2/6*, and *fabG*) were identified as involved in the synthesis of unsaturated fatty acids, mainly through fatty acid desaturation. These genes are most likely important for adjusting the membrane fluidity under cold stress. In addition, it should be noted that fatty acid desaturases require Fe^2+^ ions for their activity, while low temperatures can induce a transporter gene like *feoB* (SA2830) that codes for a high affinity ferrous iron (Fe^2+^) transport protein [28]. Low temperatures also induce the production of RNA helicases (homolog of *deaD*, *hrpB*, *helY*, *rhlE*, etc.), which prevents the formation of structured nucleic acids [24]. Although it was reported that cyanobacteria can produce extracellular polymeric substances (EPS) to survive efficiently in cold environments, *Synechococcus* sp. CS-601 (SynAce01) and other strains within the clade of marine unicellular *Synechococcus* and *Prochlorococcus* normally had a reduced genome and seem to have lost most of the EPS-related genes [29]. Similarly, none of the homologs of ice binding proteins (IBPs) have been identified. Therefore, SynAce01 cold adaptation is likely to originate from an alternative, potentially novel mechanism. Further studies are required to elucidate molecular adaptations of the strain to low temperatures.

The sigma factors of RNA polymerase play central roles in the acclimation of bacteria to different environmental conditions and can lead to a different transcription pattern when one sigma factor in the RNA polymerase holoenzyme is replaced by another [26]. Four genes encoding *rpoD* (SA1422, SA2520, SA2840, and SA2912), a Group 2 RNA polymerase sigma factor, were observed in the SynAce01 genome. The cold-induced *rpoD* is the only sigma factor abundant in the dark [30], suggesting its key role in transcription regulation in the periods of insufficient light in addition to cold in Antarctica.

Under cold stress, antisense transcription may cause a serious additional problem together with difficulties in the maintenance of a proper RNA secondary structure and loss of speed, efficiency, and fidelity of transcription and translation [26]. In eubacteria, *nusG*, a cofactor of Rho transcriptional terminator, functions in combination with histone-like nucleoid-structuring protein H-NS and Rho-dependent transcriptional terminators to diminish genome-wide antisense transcription [31]. Thus, the activation of *nusA/nusB/nusG/rho* (SA2091, SA1656, SA1342, and SA2022, respectively) expression may analogously help SynAce01 silence the global antisense transcription for survival in perennial freezing environment.

The *rpsU* gene (SA2044) for 30S ribosomal subunit protein S21 may play a role in the acclimation of the translational apparatus to cold stress, implicated by a previous report that *rpsU* was induced 10-fold by cold stress in *Synechocystis* [32]. It is known that variation in the amounts of some ribosomal proteins, such as cold-induced 50S ribosomal proteins L20 and L11 and 30S protein S12 may contribute to a fine-tuning of ribosome function and, in particular, ribosome selectivity for distinct transcripts [26]. In addition, a ribosome chaperone trigger factor (*Tig*, SA1599) can support early folding events and prevents misfolding and aggregation of proteins. The *smpB* gene (SA1659) was found in the SynAce01 genome, which encodes the protein that is required to rescue ribosomes stalled on defective messages [33].

It is known that many genes in bacteria were induced by cold shock [24]. The numbers of BLAST hits in the SynAce01 genome for genes implicated in cold shock response as compared to the three reference strains are shown in Table 2. All genes listed here were present in all genomes except for *deaD* and *desA*, which were absent from PCC7942, and *mtnA*, which was absent from MED4. On the other hand, there is no clear variation of copy numbers regarding cold stress genes between the four genomes. This evident absence of differentiation of cold shock genes between the Antarctic strain and mesophilic strains could be ascribed to the tendency of polar cyanobacteria to be psychrotrophs rather than psychrophiles. This speculation on SynAce01 was supported by its maximal growth temperature as high as 29.5 °C that was far higher than the low ambient temperature that it is likely to suffer in Antarctica. Further, similar results were found between cyanobacterial strains isolated from Antarctica and temperate lineages [9].

Cold adaptation is also associated with different types of anti-stress mechanisms. It was reported that compatible solutes, like glycine betaine act as osmolytes, contributing to psychrotolerance of a microorganism [34]. In the CS-601 (SynAce01) genome, more than one-hundred transporter-coding genes were found, indicating that the abundant gene repertoire of these transporters was probably responsible for the uptake osmolytes in order to adapt to the hostile environments in Antarctica. Glutathione maintains cell redox homeostasis also protects membrane lipids from the oxidative stress induced at cold temperatures [34]. Glutathione synthase (*gshB*, SA1410) was encoded in the SynAce01 genome, and two key genes involved in the cycle of glutathione were also found: glutathione peroxidase (*gpx*, SA1550) and glutathione reductase (*gor*, SA1091), thereby indicating that glutathione may facilitate psychrotolerance of the strain.

The acclimation of cyanobacteria to a cold environment involves a wide range of proteins related to cell modification of membrane lipids, transcription and translation regulation, and various cold-induced proteins. Therefore, it is difficult to elucidate the cold adaptation solely from the perspective of genomics. Further investigations are necessary to verify these speculations using RNA-Seq and DNA-microarray for gene expression, and random or targeted knock-out for gene function determination.

### 2.5. Strategy for Living in Oligotrophic Environment

Oligotrophic environments such as Ace Lake require its inhabitants to have special biological apparatus for survival. The CO_2_-concentrating-mechanism (CCM) that Cyanobacteria have evolved is known as a significant environmental adaptation to immensely improve the efficiency of CO_2_ fixation. CCM facilitates cyanobacterial cells in achieving a satisfactory rate of CO_2_ fixation by its active transport and accumulation as inorganic carbon (Ci: CO_2_ and HCO_3_^−^) [35]. It was reported that in cyanobacteria gaseous CO_2_-uptake systems were based on NADPH dehydrogenase (NDH-1) complexes [35]. In *Synechococcus* sp. CS-601 (SynAce01) genome, there are a number of NDH-1 genes that are present as single copies, namely *ndhB* (SA0666), *ndhM* (SA0963), *ndhAIGE* (SA0977-SA0980), *ndhH* (SA1000), *ndhCKJ* (SA1158-SA1160), *ndhN* (SA1870), and *ndhL* (SA2825); meanwhile, multiple copies were observed in *ndhD* (three copies: SA0968, SA2602, and SA2796) and *ndhF* (two copies: SA0967 and SA2600). This result is consistent with previous findings that a large diversity exists in ndhD and ndhF proteins [36]. As part of the CO_2_ uptake systems, the chpX/Y proteins are involved in enabling the CO_2_-uptake activity of the NDH-1 complex [37]. The gene cluster *ndhF4*-*ndhD4*-*chpX* (SA2600, SA2602, and SA2603, respectively) was detected in CS-601 genome and might code for constitutively expressed NDH-1_4_ complex involved in low-affinity CO_2_ uptake as indicated by phenomic evidence in previous studies [37,38]. Similar clusters were found in PCC7942 (which also possess the NDH-1_3_ complex) and WH8201, but not in MED4, which is in accord with the current conclusion that the *Prochlorococcus* species lack the capacity for active CO_2_ uptake due to the absence of either the low-affinity or high affinity NDH-1_3/4_ specific genes [39].

In addition to CO_2_ uptake systems, the genome analysis suggested that the Antarctic strain may have several types of HCO_3_^−^ uptake systems. First, two genes (SA1106 and SA2596) were detected as homologs of a low affinity, high flux, Na^+^-dependent HCO_3_^−^ transporter (*BicA*) [40]. The two genes showed the highest protein similarities with marine WH8102 (64% and 69%, respectively). Although the *BicA* transporter of WH8102 had a relatively low rate of HCO_3_^−^ uptake [40], it might be adequate for oceanic cyanobacteria which had less demand for carbon gain due to typically slow growth (one doubling per day or less), and carbon gain is light-limited in the euphotic zone. Second, marine cyanobacteria seem to not possess the high affinity HCO_3_^−^ transporter (encoded by *cmpABCD*, a traffic ATPase) that is present in many freshwater species. But, homologs of *cmpA* and *cmpB* (SA1018 and SA2620) were detected in the SynAce01 genome, exhibiting protein similarities of 63% and 23%, respectively, to that of PCC7942. Similar to *cmpA* of PCC7942, the cmpA of SynAce01 may analogously play a role in collecting HCO_3_^−^ and passing it onto the transporter [41], while the cmpB, with other ABC transporters, thereby form a transport path through the membrane [35]. Although extrinsic proteins—cmpC and cmpD—consume ATP to transport HCO_3_^−^ by allosteric regulation [35] and both were absent from the SynAce01 genome and marine cyanobacteria; this may be related to a potential strategy of employing the electrochemical driving force that is associated with maintaining a mandatory standing Na^+^ gradient (inwardly directed) for energization of uptake, rather than using ATP as a direct energy source for pumping [42]. It is also interesting to speculate that CS-601 strain has acquired *cmpAB* from a β-cyanobacterium during evolutionary course. Third, the SynAce01 genome possessed a homolog (SA0748) of *sbtA*, an inducible, high affinity Na^+^-dependent HCO_3_^−^ transporter [43]. Weak homologs of *sbtA* were present among CS-601, PCC7942, and MED4, and it is not yet clear whether the divergent forms are able to transport HCO_3_^−^. Moreover, another transporter of HCO_3_^−^ (SA1754) was detected to be a homolog of *ictB*, an HCO_3_^−^ transporter identified in PCC7942 [44]. The transporters mentioned above are expression-regulated under Ci limitation [35].

The genome of *Synechococcus* sp. CS-601 (SynAce01) has ABC-type substrate-binding proteins for phosphate and phosphonate. The strain is also able to obtain urea from the environment since the *urtABCDE* transport system and urease cluster *ureABCDEFG* were detected in the genome. A similar cluster was present in marine cyanobacteria WH8102 and MED4, while the freshwater PCC7942 lacks most of these urea-related genes. These results reinforced the importance of these transporters as a source for cyanobacterial growth in oligotrophic marine environments. Although inorganic nitrogen and phosphorus are often quite limiting in the marine environment, an alternative strategy may be conducted by the Antarctic strain to supplement the deficiency of essential uptake. For example, genes for an amino acid transporter (e.g., SA1517 and SA1697) were found, suggesting the capability of the Lake Ace isolate to use these ubiquitous compounds in the marine environment; SynAce01 also has multiple genes for a phosphate transporter (e.g., SA0762 and SA2697). Additionally, the strain has genes for phosphonate transporter (SA0763) and alkaline phosphatase (SA1541), suggesting that an Antarctic isolate could obtain phosphate from other organic phosphorus sources in the surrounding environment. The genome analysis above implied that CS-601 might not depend solely on inorganic forms of nutrients in order to survive in the barren environments. The above results are consistent with the previous finding that marine cyanobacteria use organic nitrogen and phosphorus sources more often than freshwater cyanobacteria [19].

Linker polypeptides are necessary for the correct assembly of phycobiliprotein in phycobilisome rods [19]. Phycoerythrin-associated linker protein *cpeS* was present in all four strains, while *cpcG* and *cpcT* were found only in SynAce01, WH8102, and PCC7942, and *cpcF* only in SynAce01 and PCC7942. Homologs of *cpcC* and *cpcD*, encoding two types of phycocyanin-associated linker protein in freshwater cyanobacteria, were absent in the Antarctic strain, but interestingly there was an additional pair of R-phycocyanin alpha and beta subunits to complement C-phycocyanin alpha and beta subunits, indicating a potentially new way of adapting the photosystem composition to changing environmental conditions. These genome results implicated a basis for the interpretation of absorbance spectra of the strain, which is that genes related to phycoerythrin (blue light-absorbing) are probably crucial, since blue light is particularly important for strains to survive in the oligotrophic marine environment [45]. In addition, the *Synechococcus* sp. CS-601 (SynAce01) genome lacks homologs of *nblA* and *nblB* which function in the degradation of phycobilisomes during nutrient stress in cyanobacteria [46], indicating that phycobilisome degradation may not take place or be controlled by other genes.

### 2.6. Salinity Adaptation Strategy

Cyanobacterial *mrp*-like clusters have been reported to be involved in salt stress tolerance and CO_2_ deficiency-induced expression [47]. An *mrp* homolog gene cluster (*mrpBCDEFG*) was found in the SynAce01 genome, which, together with other sodium/proton antiporters (SA0930, SA2403, and SA2570), might function as an Na^+^/H^+^ antiporter for salinity stress tolerance. It is interesting that an *mrp* gene cluster was absent from marine WH8102 and MED4, but present in the freshwater PCC7942. Salinity adaptation also relies on the active transport of nutrients in exchange of sodium, *via* cotransport symporters and translocation systems [48]. Several symporters were present in the genome of the Antarctic strain, including sulfate/sodium (SA1997), glutamate/sodium (SA2039), and bile acids/sodium (SA0197, SA0395, and SA1247). Moreover, the *kefB* (SA1466) and *trkA/G* (SA1995 and SA2610) transporters may help the strain accumulate potassium as a pH regulator to maintain pH homeostasis in cells, as indicated by previous report that these transporters were responsible for salt adaptation [49].

It was reported previously that freshwater *Synechococcus* sp. PCC7942 became more halotolerant after being genetically engineered to synthesize glycine betaine [50]. This result indicated glycine betaine and related compounds act as osmolytes and may play an important role in salinity adaptation. In the SynAce01 genome, a glycine/betaine ABC transporter (SA2274) was found as along with genes predicted to synthesize glycine betaine from choline (choline dehydrogenase, SA2379; oxidoreductase, SA0564). Glycerol is another important osmoprotectant, which is ubiquitous in saline habitats and mainly produced by the unicellular green algae *Dunaliella* [51]. In its catabolic degradation, the pathway, involving glycerol kinase (*glpK*, SA0875) and glycerol-3-phosphate dehydrogenase (*glpA*, SA0877; *gpsA*, SA2737), was found in the SynAce01 genome.

In summary, the basic mechanism of salinity adaptation is to prevent the inorganic salts from entering the cell and to utilize organic osmolytes to balance the high salinity of the environment. Further investigations are required to elucidate the exact mechanism of salinity adaptation, such as transcriptomics and characterization of amino acid composition.

### 2.7. Phylogenetic Analysis

**Figure 4:**
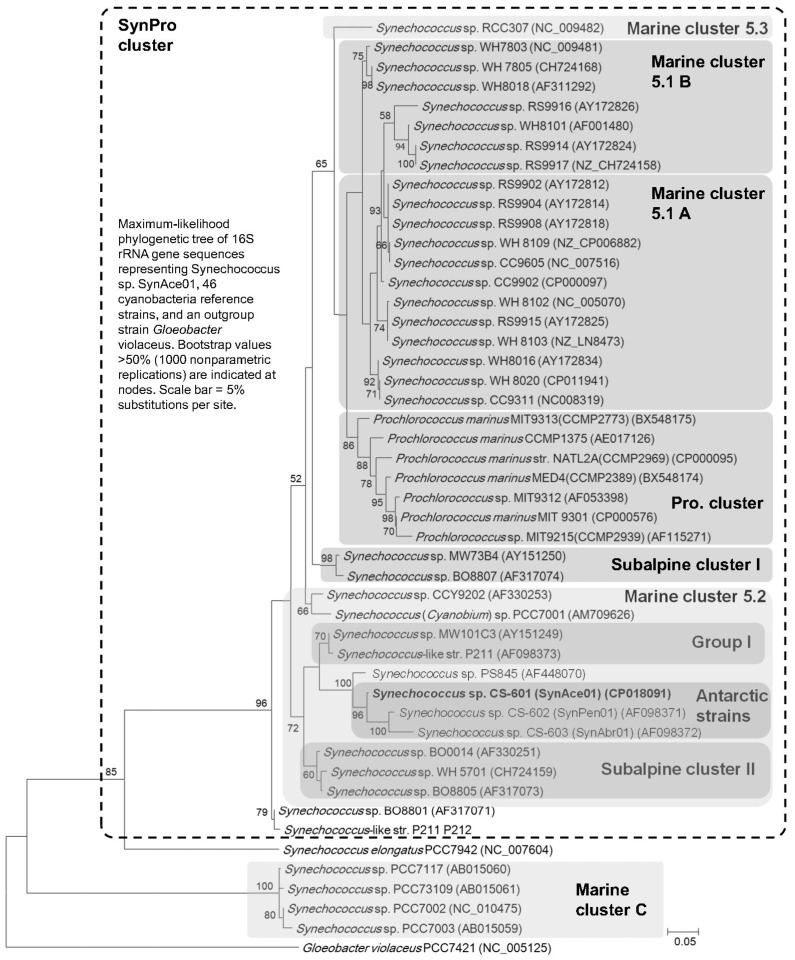
Maximum-likelihood phylogenetic tree of 16S rRNA gene sequences representing *Synechococcus* sp. SynAce01, 46 cyanobacteria reference strains, and an outgroup strain—*Gloeobacter violaceus*. Bootstrap values >50% (1000 nonparametric replications) are indicated at nodes. Scale bar = 5% substitutions per site. For taxonomic, temperature, and habitat characteristics of these picocyanobacteria please consult Table S4.

Phylogenetic analysis using the 16S rRNA gene revealed that *Synechococcus* sp. CS-601 (SynAce01) is positioned within a broad SynPro [52] clade of cyanobacteria (Figure 4), more specifically, in poorly represented and described parts of the clade that include Marine cluster 5.2 [53,54], Baltic Sea isolates [55], and clusters traditionally associated with freshwater lakes such as Subalpine cluster II and group I [56]. Compared to Marine cluster 5.1A and 5.1B (Figure 4 and Table S4 (strain details)), this section of the phylogenetic tree contains strains with comparatively the lowest amount of information available (Table S4). Unsurprisingly, the three Antarctic strains isolated from Vestfold Hills lakes cluster together. They show the closest phylogenetic relationship with *Synechococcus* sp. PS845, a poorly described strain of marine origin isolated from the coastal region of Russia [57]. Two other strains in which Antarctic strains also show significant similarity on the basis of 16S rRNA gene are more informative: *Synechococcus* sp. P211, an Arctic strain isolated from High Arctic territories of Canada [7,58] and *Synechococcus* sp. MW101C3, isolated from a deep subalpine, oligomesotrophic lake—Mondsee (Austria) [57]. Both of these strains show environmental similarity to that of Vestford Hills where SynAce01 was isolated i.e. are cold, oligotrophic environments, albeit freshwater. More broadly, all the strains described above share some common characteristics. They are likely to be euryhaline isolates from coastal regions of the sea or lakes (both freshwater and saline), and predominantly from cold environments (Antarctica, Arctic, Baltic Sea, subalpine lakes). The exception to this pattern is *Cyanobium* sp. PCC7001, which has a relatively high growth temperature (Table S4); it clearly branches out of other members that were included in the Marine cluster 5.2 in this study. *Cyanobium* sp. PCC7001 and related strains are often included in Marine cluster 5.2 in other studies. These studies, however, typically lack strains we have included in this analysis resulting in clustering the *Cyanobium* sp. PCC7001 and *Synechococcus sp.* WH5701 together. This entire part of the SynPro clade along with introduction of new species has been debated for a while [59], and we believe that a larger representation of strains will be required for comprehensive analysis of the Marine cluster 5.2 in the future. Hopefully, sequencing the *Synechococcus sp.* SynAce01 genome can contribute to expanding knowledge about these strains. To date, *Synechococcus sp.* WH5701 and *Synechococcus sp.* SynAce01 are the only representatives of this cluster sequenced genomes.

## 3. Conclusion

The overall genome size of *Synechococcus* sp. CS-601 (SynAce01) and its GC content is typical when compared to other members of Synechococcaceae. The strain belongs to most underrepresented clades of cyanobacteria, and its genome sequence will be valuable for genomic, taxonomic, phylogenetic, and functional studies of strains from cold, oligotrophic environments. Genome analysis reveals two patterns of adaptation to the inhospitable Antarctic environment. Its adjustment to the saline and oligotrophic environment is conferred by an abundance of nutrient and osmolyte transporters and multiple forms of light harvesting components, some of them likely acquired through horizontal gene transfer. This suggests that pulse evolutionary events were important in gaining these traits. The expanded availability of key nutrients and energy could have allowed an ancestral strain to colonise inhospitable environmental niches. Adaptations of the strain to low temperatures are more subtle and are likely to be a result of prolonged exposure to low temperatures and slow adaptation of key functional components to the new environment. More detailed functional studies are required to verify these findings.

## 4. Materials and Methods

### 4.1. Bacterial Strain and DNA Extraction

The bacterial strain used in the present study was *Synechococcus* sp. CS-601 (SynAce01) (referred to as SynAce01 or CS-601), which was isolated from Ace Lake, Antarctic, by Lynne Rankin (Powell) in 1992 [6]. The cells of strain CS-601 were cultured in GSe medium + soil extract [60], and grown in a controlled climate chamber for three weeks at 10 °C under a photoperiod of 24-h light (2.5 umol photons PAR m^−2^·s^−1^). The total genomic DNA was extracted and purified using a bacterial genomic DNA isolation kit (Generay, Shanghai, China) according to the manufacturer’s instructions. Purified genomic DNA was subjected to gel electrophoresis and spectrophotometric measurements for quality and quantity assessment, respectively.

### 4.2. Genome Sequencing and De Novo Assembly

The whole-genome sequencing of SynAce01 was performed using two sequencing strategies: PacBio RS II and Illumina HiSeq 4000. Two SMRT cells were used for PacBio sequencing and yielded 46,415 adapter-trimmed reads (subreads) with an average read length of approximately 7 kbp, which corresponded to 120-fold coverage. De novo assembly was performed using the hierarchical genome assembly process (HGAP) method implemented in SMRT analysis v2.3.0 [61], generating a single contig. Illumina sequencing of SynAce01 generated a total of 1,448,782 filtered paired-end reads (clean data), providing approximately 150-fold coverage of the genome. The clean data was assembled into contigs using SOAPdenovo v2.04 [62] with default parameters. Based on the contigs from SOAPdenovo assembler, the contigs derived from the HGAP method were comparatively examined to determine their continuity with one another and were concatenated into one closed circular chromosome. The genome obtained was mapped by Illumina Hiseq reads to correct any assembly and sequence errors using SOAPsnp [63], SOAPindel and Genome Analysis Toolkit (https://software.broadinstitute.org/gatk/). The circular chromosome was further confirmed by SSPACE-LongRead [64] based on the subreads derived from PacBio system. Final genome has been deposited in Genbank with an accession number CP018091.

### 4.3. Genome Annotation

The genome of SynAce01 was annotated using a customized pipeline. In the pipeline, CDSs were identified by GeneMarks v4.6b [65], and genes for tRNAs and rRNAs were predicted by tRNAscan-SE v1.3.1 [66] and RNAmmer v1.2 [67]. The predicted CDSs were functionally annotated based on homology searches against the public databases, including NR, KEGG, COG, Swiss-Prot, and TrEMBL. These data sources were combined to claim the annotation of each predicted protein. Besides, the genome sequence was automatically annotated using the NCBI PAPPC pipeline [68]. The annotations derived from the two pipelines were compared and some of the results were manually curated. The insertion sequence (IS) was detected and annotated by ISsaga [69]. Prophage regions were predicted by PHASTER [70]. CRISPR loci were detected using CRISPRFinder server [71]. The outputs of blast searching against the NCBI nr protein database were imported into BLAST2GO V5.1 [72] for GO term mapping. The results of BLAST2GO analysis were submitted to the WEGO [73] for GO classification under the biological process, molecular function and cellular component ontologies. The circular plot of SynAce01 genome was produced in Circos v0.68 [74].

### 4.4. Comparative Genome Analysis

The annotated genome sequences of three reference genomes were downloaded from NCBI and included in the comparative genome analysis. The three reference genomes were *Synechococcus* sp. WH8102 (NC_005070), *Synechococcus elongatus* PCC 7942 (NC_007604), and *Prochlorococcus marinus* MED4 (NC_005072) (referred to as WH8102, PCC7942 and MED4, respectively). To compare the gene context, all-against-all BLASTP alignments were performed between SynAce01 and each reference strain. The BLASTP alignments were conducted using the following thresholds, *E*-value cut-off of 1E-5 and ≥30% identity. The customized Venn diagram was drawn using an online tool to exhibit the orthologous and unique genes of the four strains.

### 4.5. Phylogenetic Analysis

A Maximum-likelihood (ML) phylogenetic analysis was performed using complete 16S rRNA gene sequences of SynAce01 and 46 cyanobacteria reference strains retrieved from GenBank. The 16S rRNA gene sequence of *Gloeobacter violaceus* was also included in the phylogenetic analysis as an outgroup. A multiple alignment of sequences was generated using MUSCLE as implemented in MEGA 6 [75]. ML analysis of 16S gene sequences was carried out using PhyML v3.0 [76]. Parameters used in PhyML were set as described by Tang et al. [5].

## Figures and Tables

**Table 1:** Genome characteristics of strains used in this study.

	*Synechococcus* sp. CS-601 (SynAce01)	*Synechococcus* sp. WH8102	*P. marinus* MED4	*S. elongatus* PCC7942
Isolation source	Ace Lake, Antarctic	Sargasso Sea 22.495° N 65.6° W	Eastern Mediterranean Sea 35° N 20°E (approx.)	California USA
Habitat	Saline lake	Tropical Seawater	Shallow seawater (depth 5M) high light adapted strain	Freshwater
Temperature range	−17–29.5	12–30	17–27	25–45
Genome status	Complete	Complete	Complete	Complete
Genome size (bp)	2,750,634	2,434,428	1,657,990	2.74227
GC content (%)	63.92	59.40	30.80	55.46
CDSs	2984	2513	1790	2685
rRNA operons	2	2	1	2
tRNAs	44	43	37	44
Other RNA	4	4	4	4
Accession no.	CP018091	NC_005070	NC_005072	NC_007604

**Table 2:** Numbers of BLASTP hits for genes implicated in bacterial cold shock response in all strains used.

Gene ^1^	Description	Number of BLASTP Hits			
		*Synechococcus* sp. SynAce01	*Synechococcus* sp. WH8102	*P. marinus* MED4	*S. elongatus* PCC 7942
*aceF*	Pyruvate dehydrogenase E2 component	1	1	1	1
*csp*-family	Cold shock protein/cold-inducible RNA chaperone	2	3	2	3
*deaD*	DEAD-like RNA helicase	2	2	1	0
*desA*	Fatty acid desaturase	2	2	2	0
*dnaA*	Replication initiation protein	1	1	1	1
*dnaJ*	Molecular chaperone	8	7	5	5
*gyrA*	DNA gyrase subunit A	1	2	2	2
*infA*	Translation initiation factor IF-1	1	1	1	1
*infB*	Translation initiation factor IF-2	1	1	1	1
*infC*	Translation initiation factor IF-3	1	1	1	1
*mtnA*	Translation initiation factor IF-2B subunit alpha	1	1	0	1
*nusA*	Transcription termination factor	1	1	1	1
*pdhA*	Pyruvate dehydrogenase E1 subunit alpha	1	1	2	1
*pdhB*	Pyruvate dehydrogenase E1 subunit beta	1	1	1	1
*rbfA*	Ribosome-binding factor A	1	1	1	1
*recA*	Recombination and DNA repair	1	1	1	1
*tig*	Protein-folding chaperone	1	1	1	1

^1^ Genes implicated in bacterial cold shock response were derived from Barria et al. [24].

## Supplementary Materials

Supplementary materials can be found at http://www.mdpi.com/1422-0067/20/1/152/s1.

## References

[1] Pittera J., Humily F., Thorel M., Grulois D., Garczarek L., Six C. (2014). Connecting thermal physiology and latitudinal niche partitioning in marine *Synechococcus*. ISME J..

[2] Buitenhuis E.T., Li W.K.W., Vaulot D., Lomas M.W., Landry M.R., Partensky F., Karl D.M., Ulloa O., Campbell L., Jacquet S. (2012). Picophytoplankton biomass distribution in the global ocean. Earth Syst. Sci. Data.

[3] Pittera J., Partensky F., Six C. (2016). Adaptive thermostability of light-harvesting complexes in marine picocyanobacteria. ISME J..

[4] Ward D.M., Castenholz R.W. (2000). Cyanobacteria in geothermal habitats. The Ecology of Cyanobacteria.

[5] Tang J., Jiang D., Luo Y., Liang Y., Li L., Shah M.M.R., Daroch M. (2018). Potential new genera of cyanobacterial strains isolated from thermal springs of western Sichuan, China. Algal Res..

[6] Powell L. (2005). Ecology of a novel *Synechococcus* clade occurring in dense populations in saline Antarctic lakes. Mar. Ecol. Prog..

[7] Vincent W.F., Bowman J.P., Rankin L.M., McMeekin T.A. (2000). Phylogenetic diversity of picocyanobacteria in Arctic and Antarctic ecosystems. Microbial Biosystems: New Frontiers, Proceedings of the 8th International Symposium on Microbial Ecology.

[8] Chrismas N., Anesio A.M., Sánchezbaracaldo P. (2018). The future of genomics in polar and alpine cyanobacteria. FEMS Microbiol. Ecol..

[9] Chrismas N.A.M., Barker G., Anesio A.M., Sánchezbaracaldo P. (2016). Genomic mechanisms for cold tolerance and production of exopolysaccharides in the Arctic cyanobacterium *Phormidesmis priestleyi* BC1401. BMC Genom..

[10] Fuller C.W., Middendorf L.R., Benner S.A., Church G.M., Harris T., Huang X., Jovanovich S.B., Nelson J.R., Schloss J.A., Schwartz D.C. (2009). The challenges of sequencing by synthesis. Nat. Biotechnol..

[11] Dohm J.C., Lottaz C., Borodina T., Himmelbauer H. (2008). Substantial biases in ultra-short read data sets from high-throughput DNA sequencing. Nucleic Acids Res..

[12] Eid J., Fehr A., Gray J., Luong K., Lyle J., Otto G., Peluso P., Rank D., Baybayan P., Bettman B. (2010). Real-time DNA sequencing from single polymerase molecules. Methods Enzymol..

[13] Rasko D.A., Webster D.R., Sahl J.W., Bashir A., Boisen N., Scheutz F., Paxinos E.E., Sebra R., Chin C.S., Iliopoulos D. (2011). Origins of the *E. coli* strain causing an outbreak of hemolytic–uremic syndrome in Germany. N. Engl. J. Med..

[14] Chin C.S., Sorenson J., Harris J.B., Robins W.P., Charles R.C., Jeancharles R.R., Bullard J., Webster D.R., Kasarskis A., Peluso P. (2011). The origin of the Haitian *Cholera* outbreak strain. N. Engl. J. Med..

[15] Bashir A., Klammer A.A., Robins W.P., Chin C.S., Webster D., Paxinos E., Hsu D., Ashby M., Wang S., Peluso P. (2012). A hybrid approach for the automated finishing of bacterial genomes. Nat. Biotechnol..

[16] Okoniewski M.J., Meienberg J., Patrignani A., Szabelska A., Matyas G., Schlapbach R. (2013). Precise breakpoint localization of large genomic deletions using PacBio and Illumina next-generation sequencers. BioTechniques.

[17] Wu Z., Gui S., Quan Z., Pan L., Wang S., Ke W., Liang D., Ding Y. (2014). A precise chloroplast genome of *Nelumbo nucifera* (Nelumbonaceae) evaluated with Sanger, Illumina MiSeq, and PacBio RS II sequencing platforms: Insight into the plastid evolution of basal eudicots. BMC Plant Biol..

[18] Mikheeva L.E., Karbysheva E.A., Shestakov S.V. (2013). The role of mobile genetic elements in the evolution of cyanobacteria. Russ. J. Genet..

[19] Palenik B., Brahamsha B., Larimer F.W., Land M., Hauser L., Chain P., Lamerdin J., Regala W., Allen E.E., Mccarren J. (2003). The genome of a motile marine *Synechococcus*. Nature.

[20] Marraffini L.A., Sontheimer E.J. (2010). CRISPR interference: RNA-directed adaptive immunity in bacteria and archaea. Nat. Rev. Genet..

[21] Marraffini L.A., Sontheimer E.J. (2008). CRISPR interference limits horizontal gene transfer in *staphylococci* by targeting DNA. Science.

[22] Blindauer C.A. (2008). Zinc-handling in cyanobacteria: An update. Chem. Biodivers..

[23] Saita E., Albanesi D., De M.D. (2016). Sensing membrane thickness: Lessons learned from cold stress. Biochim. Biophys. Acta.

[24] Barria C., Malecki M., Arraiano C.M. (2013). Bacterial adaptation to cold. Microbiology.

[25] Los D.A., Murata N. (1999). Responses to cold shock in cyanobacteria. J. Mol. Microbiol. Biotechnol..

[26] Sinetova M.A., Los D.A. (2016). New insights in cyanobacterial cold stress responses: Genes, sensors, and molecular triggers. Biochim. Biophys. Acta.

[27] Kappell A.D., van Waasbergen L.G. (2007). The response regulator *RpaB* binds the high light regulatory 1 sequence upstream of the high-light-inducible *hliB* gene from the cyanobacterium *Synechocystis* PCC 6803. Arch. Microbiol..

[28] Hantke K. (2003). Is the bacterial ferrous iron transporter FeoB a living fossil?. Trends Microbiol..

[29] Pereira S.B., Mota R., Vieira C.P., Vieira J., Tamagnini P. (2015). Phylum-wide analysis of genes/proteins related to the last steps of assembly and export of extracellular polymeric substances (EPS) in cyanobacteria. Sci. Rep..

[30] Tuominen I., Tyystjärvi E., Tyystjärvi T. (2003). Expression of primary sigma factor (PSF) and PSF-like sigma factors in the cyanobacterium *Synechocystis* sp. strain PCC 6803. J. Bacteriol..

[31] Peters J.M., Mooney R.A., Grass J.A., Jessen E.D., Tran F., Landick R. (2012). *Rho* and *NusG* suppress pervasive antisense transcription in *Escherichia coli*. Genes Dev..

[32] Sato N., Tachikawa T., Wada A., Tanaka A. (1997). Temperature-dependent regulation of the ribosomal small-subunit protein S21 in the cyanobacterium *Anabaena variabilis* M3. J. Bacteriol..

[33] Karzai A.W., Susskind M.M., Sauer R.T. (1999). *SmpB*, a unique RNA-binding protein essential for the peptide-tagging activity of *SsrA* (tmRNA). Embo J..

[34] Tanizawa Y., Tohno M., Kaminuma E., Nakamura Y., Arita M. (2015). Complete genome sequence and analysis of *Lactobacillus hokkaidonensis* LOOC260^T^, a psychrotrophic lactic acid bacterium isolated from silage. BMC Genom..

[35] Price G.D., Badger M.R., Woodger F.J., Long B.M. (2008). Advances in understanding the cyanobacterial CO_2_-concentrating-mechanism (CCM): Functional components, Ci transporters, diversity, genetic regulation and prospects for engineering into plants. J. Exp. Bot..

[36] Price G.D., Sültemeyer D., Klughammer B., Ludwig M., Badger M.R. (1998). The functioning of the CO_2_ concentrating mechanism in several cyanobacterial strains: A review of general physiological characteristics, genes, proteins, and recent advances. Can. J. Bot..

[37] Maeda S.I., Badger M.R., Price G.D. (2010). Novel gene products associated with NdhD3/D4-containing NDH-1 complexes are involved in photosynthetic CO_2_ hydration in the cyanobacterium, *Synechococcus* sp. PCC7942. Mol. Mircrobiol..

[38] Shibata M., Ohkawa H., Kaneko T., Fukuzawa H., Tabata S., Kaplan A., Ogawa T. (2001). Distinct constitutive and low-CO_2_-induced CO_2_ uptake systems in cyanobacteria: Genes involved and their phylogenetic relationship with homologous genes in other organisms. Proc. Natl. Acad. Sci. USA.

[39] Badger M.R., Price G.D. (2003). CO_2_ concentrating mechanisms in cyanobacteria: Molecular components, their diversity and evolution. J. Exp. Bot..

[40] Price G.D., Woodger F.J., Badger M.R., Howitt S.M., Tucker L. (2004). Identification of a SulP-type bicarbonate transporter in marine cyanobacteria. Proc. Natl. Acad. Sci. USA.

[41] Omata T., Price G.D., Badger M.R., Okamura M., Gohta S., Ogawa T. (1999). Identification of an ATP-binding cassette transporter involved in bicarbonate uptake in the Cyanobacterium *Synechococcus* sp. strain PCC7942. Proc. Natl. Acad. Sci. USA.

[42] Bryant D.A. (2003). The Beauty in Small Things Revealed. Proc. Natl. Acad. Sci. USA.

[43] Shibata M., Katoh H., Sonoda M., Ohkawa H., Shimoyama M., Fukuzawa H., Kaplan A., Ogawa T. (2002). Genes essential to sodium-dependent bicarbonate transport in cyanobacteria: Function and phylogenetic analysis. J. Biol. Chem..

[44] Bonfil D.J., Ronen-Tarazi M., Sültemeyer D., Lieman-Hurwitz J., Schatz D., Kaplan A. (1998). A putative HCO_3_^−^ transporter in the cyanobacterium *Synechococcus* sp. strain PCC7942. FEBS Lett..

[45] Platt T., Li W.K.W. (1986). Photosynthetic Picoplankton.

[46] Dolganov N., Grossman A.R. (1999). A polypeptide with similarity to phycocyanin α-subunit phycocyanobilin lyase involved in degradation of phycobilisomes. J. Bacteriol..

[47] Fukaya F., Promden W., Hibino T., Tanaka Y., Nakamura T., Takabe T. (2009). An mrp-Like cluster in the halotolerant cyanobacterium *Aphanothece halophytica* functions as a Na^+^/H^+^ antiporter. Appl. Environ. Microbiol..

[48] López-Pérez M., Ghai R., Leon M.J., Rodríguez-Olmos Á., Copa-Patiño J.L., Soliveri J., Sanchez-Porro C., Ventosa A., Rodriguez-Valera F. (2013). Genomes of *Spiribacter*, a streamlined, successful halophilic bacterium. BMC Genom..

[49] Mongodin E.F., Nelson K.E., Daugherty S., DeBoy R.T., Wister J., Khouri H., Weidman J., Walsh D.A., Papke R.T., Sanchez Perez G. (2005). The genome of *Salinibacter ruber*: Convergence and gene exchange among hyperhalophilic bacteria and archaea. Proc. Natl. Acad. Sci. USA.

[50] Nomura M., Ishitani M., Takabe T., Rai A.K. (1995). *Synechococcus* sp. PCC7942 transformed with *Escherichia coli bet* genes produces glycine betaine from choline and acquires resistance to salt stress. Plant Physiol..

[51] Bardavid R.E., Khristo P., Oren A. (2008). Interrelationships between *Dunaliella* and halophilic prokaryotes in saltern crystallizer ponds. Extremophiles.

[52] Blank C.E., Sánchez-Baracaldo P. (2010). Timing of morphological and ecological innovations in the cyanobacteria—A key to understanding the rise in atmospheric oxygen. Geobiology.

[53] Scanlan D.J., Ostrowski M., Mazard S., Dufresne A., Garczarek L., Hess W.R., Post A.F., Hagemann M., Paulsen I., Partensky F. (2009). Ecological genomics of marine picocyanobacteria. Microbiol. Mol. Biol. Rev..

[54] Cabelloyeves P.J., Haromoreno J.M., Martincuadrado A.B., Ghai R., Picazo A., Camacho A., Rodriguezvalera F. (2017). Novel *Synechococcus* genomes reconstructed from freshwater reservoirs. Front. Microbiol..

[55] Haverkamp T.H.A., Schouten D., Doeleman M., Wollenzien U., Huisman J., Stal L.J. (2009). Colorful microdiversity of *Synechococcus* strains (picocyanobacteria) isolated from the Baltic Sea. ISME J..

[56] Callieri C., Coci M., Corno G., Macek M., Modenutti B., Balseiro E., Bertoni R. (2013). Phylogenetic diversity of nonmarine picocyanobacteria. FEMS Microbiol. Ecol..

[57] Crosbie N.D., Pöckl M., Weisse T. (2003). Dispersal and phylogenetic diversity of nonmarine picocyanobacteria, inferred from 16S rRNA gene and cpcBA-intergenic spacer sequence analyses. Appl. Environ. Microbiol..

[58] Vézina S., Vincent W.F. (1997). Arctic cyanobacteria and limnological properties of their environment: Bylot Island, Northwest Territories, Canada (73° N, 80° W). Polar Biol..

[59] Ernst A., Becker S., Wollenzien U.I.A., Postius C. (2003). Ecosystem-dependent adaptive radiations of picocyanobacteria inferred from 16S rRNA and ITS-1 sequence analysis. Microbiology.

[60] Iii A.R.L., Smith V.E. (1968). Chloroplast pigments of the marine dinoflagellate *Gyrodinium resplendens*. Lipids.

[61] Chin C.S., Alexander D.H., Marks P., Klammer A.A., Drake J., Heiner C., Clum A., Copeland A., Huddleston J., Eichler E.E. (2013). Nonhybrid, finished microbial genome assemblies from long-read SMRT sequencing data. Nat. Methods.

[62] Luo R., Liu B., Xie Y., Li Z., Huang W., Yuan J., He G., Chen Y., Qi P., Liu Y. (2012). SOAPdenovo2: An empirically improved memory-efficient short-read de novo assembler. Gigascience.

[63] Li R., Li Y., Fang X., Yang H., Wang J., Kristiansen K., Wang J. (2009). SNP detection for massively parallel whole-genome resequencing. Genome Res..

[64] Boetzer M., Henkel C.V., Jansen H.J., Butler D., Pirovano W. (2011). Scaffolding pre-assembled contigs using SSPACE. Bioinformatics.

[65] Besemer J., Lomsadze A., Borodovsky M. (2001). GeneMarkS: A self-training method for prediction of gene starts in microbial genomes. Implications for finding sequence motifs in regulatory regions. Nucleic Acids Res..

[66] Lowe T.M., Eddy S.R. (1997). tRNAscan-SE: A program for improved detection of transfer RNA genes in genomic sequence. Nucleic Acids Res..

[67] Lagesen K., Hallin P., Rødland E.A., Stærfeldt H.H., Rognes T., Ussery D.W. (2007). RNAmmer: Consistent and rapid annotation of ribosomal RNA genes. Nucleic Acids Res..

[68] Pruitt K.D., Tatusova T., Klimke W., Maglott D.R. (2009). NCBI Reference Sequences: Current status, policy and new initiatives. Nucleic Acids Res..

[69] Varani A.M., Siguier P., Gourbeyre E., Charneau V., Chandler M. (2011). ISsaga is an ensemble of web-based methods for high throughput identification and semi-automatic annotation of insertion sequences in prokaryotic genomes. Genome Biol..

[70] Arndt D., Grant J.R., Marcu A., Sajed T., Pon A., Liang Y., Wishart D.S. (2016). PHASTER: A better, faster version of the PHAST phage search tool. Nucleic Acids Res..

[71] Grissa I., Vergnaud G., Pourcel C. (2007). CRISPRFinder: A web tool to identify clustered regularly interspaced short palindromic repeats. Nucleic Acids Res..

[72] Conesa A., Götz S., Garcíagómez J.M., Terol J., Talón M., Robles M. (2005). Blast2GO: A universal tool for annotation, visualization and analysis in functional genomics research. Bioinformatics.

[73] Ye J., Fang L., Zheng H., Zhang Y., Chen J., Zhang Z., Wang J., Li S., Li R., Bolund L., Wang J. (2006). WEGO: A web tool for plotting GO annotations. Nucleic Acids Res..

[74] Krzywinski M.I., Schein J.E., Birol I., Connors J., Gascoyne R., Horsman D., Jones S.J., Marra M.A. (2009). Circos: An information aesthetic for comparative genomics. Genome Res..

[75] Tamura K., Stecher G., Peterson D., Filipski A., Kumar S. (2013). MEGA6: Molecular evolutionary genetics analysis version 6.0. Mol. Biol. Evol..

[76] Guindon S., Dufayard J.F., Lefort V., Anisimova M., Hordijk W., Gascuel O. (2010). New algorithms and methods to estimate maximum-likelihood phylogenies: Assessing the performance of PhyML 3.0. Syst. Biol..

