# Supplementary material for: Complete Genome Sequence and Comparative Analysis of Synechococcus sp. CS-601 (SynAce01), a Cold-Adapted Cyanobacterium from an Oligotrophic Antarctic Habitat
Source: Int J Mol Sci. 2019 Jan 3;20(1):152. doi: 10.3390/ijms20010152 (PMC6337551; doi:10.3390/ijms20010152)
Supplement: Supplementary file 1 [file ijms-20-00152-s001.zip › Supplementary Table S4.docx]

**Table S4** Taxonomic, temperature and habitat characteristics of known representatives of picocyanobacteria

| **Strain name** | **Other names** | **Strain origin and habitat** | **Accession number*** | **Taxonomic cluster** | **Cell features** | **Temperature information** | **Other remarks** | **Ref.** | |
| --- | --- | --- | --- | --- | --- | --- | --- | --- | --- |
| *Synechococcus* sp. SynAce01 | CS-601 | 68.28S, 78.11E  Antarctica, Vestfold Hills, Ace Lake  11 m depth  Salinity 30 g L^-1^ | CP018091 (G) | This study | Elongated non-motile cells  L: 1.5-1.8 µm  Pigment: T2 (with multiple types of phycocyanin CPC RPC) | T_isol_: 4.5°C  T_min_: -17 °C  T_max_: 29.5°C  T_opt_: 20°C  T_main_: 10°C | Broad light intensity: 2.5 µmol photons PAR m^-2^ s^-1^ to 300 µmol photons PAR m^-2^ s^-1^  Opt. salinities of 20-30 g kg^-1^; no growth < 10 and >50 g kg^-1^ | [1, 2]  This study | |
| *Synechococcus* sp. SynPen01 | CS-602 | 68.28S, 78.11E  Antarctica, Vestfold Hills,  Pendant Lake  6 m depth  Salinity 16.5-31.0 g L^-1^ | AF098371 (R) | This study | Elongated non-motile cells  L: 1.5-1.8 µm  Pigment: T2 | T_isol_: 0.2-0.8°C  T_min_: ND  T_max_: ND  T_opt_: ND  T_main_: 10°C |  | [1, 2] | |
| *Synechococcus* sp. SynAbr01 | CS-603 | 68.28S, 78.11E  Antarctica, Vestfold Hills,  Lake Abraxas  19 m depth  Salinity  16.8-20.3 g L^-1^ | AF098372 (R) | This study | Elongated non-motile cells  L: 1.5-1.8 µm  Pigment: T2 | T_isol_: 0.5-8.0°C  T_min_: ND  T_max_: ND  T_opt_: ND  T_main_: 10°C |  | [1, 2] | |
| *Synechococcus* sp. WH7805 | RCC1085 | 33.73N  67.50W  Sargasso Sea, pelagic marine surface water | CH724168 (D) | MarClust_5.1B_VI | Elongated cells  L: 2.0 µm  Pigment: T2 | T_isol_: 25-27.9°C  T_min_: ND  T_max_: ND  T_opt_: ND  T_main_: 22°C |  | [3–5] | |
| *Synechococcus* sp. WH8018 | RCC2373 | 41.52N 70.67W  Atlantic Ocean, Woods Hole, coastal ecosystem | AF311292 (R) | Mar.Clust_5.1B_VI | Coccoid non-motile cells  Pigment: T2 | T_isol_: ND  T_min_: ND  T_max_: ND  T_opt_: ND  T_main_: 22°C |  | [4] | |
| *Synechococcus* sp. WH7803 | RCC752, DC2, CCMP1334  NEPCC549 | 33.75N 67.50W  Sargasso Sea, pelagic marine water  25 m depth | NC_009481 (G) | MarClust_5.1B_V | Elongated non-motile cells  L: 1.0-2.0 µm  Pigment: T3a | T_isol_: 25-27.9°C  T_min_: 16°C  T_max_: 30°C  T_opt_: 28°C  T_main_: 20°C |  | [4, 6, 7]  RCC web  NCMA web | |
| *Prochlorococcus marinus* MIT9313 | CCMP2773, RCC407 | 37.50° N 68.23° W  North Atlantic Gulf stream, 135m depth | BX548175 (G) | Clust.LLIV; | Elongated, non-motile,  L: 1.2-1.6 µm  low light adapted  No PBS | T_isol_: ND  T_min_: 18°C  T_max_: 26°C  T_opt_: ND  T_main_: 20°C | Widely distributed within the 40°N to 35°S latitudinal range, restricted to the deep euphotic zone | [8, 9]  NCMA web | |
| *Prochlorococcus marinus* CCMP1375 | SS120 | 30.00° N 60.00° W  North Atlantic Sargasso Sea  Deep eutrophic zone  120m depth | AE017126 (G) |  | Elongated, non-motile,  L: 1.2-1.6 µm  low light adapted  No PBS | T_isol_: ND  T_min_: 14°C  T_max_: 27°C  T_opt_: ND  T_main_: 20°C | Grown in dim light  Present in deep waters at very low concentrations | [8, 9]  NCMA web | |
| *Prochlorococcus marinus* NATL2A | CCMP2969 | North Atlantic 39.00° N 49.30° W  Depth 10m | CP000095 (G) | Clust LLII; | Elongated, non-motile,  L: 1.2-1.6 µm  low light adapted  No PBS | T_isol_: ND  T_min_: 18°C  T_max_: 22°C  T_opt_: ND  T_main_: 20°C | Grown in dim light  Typically found in an intermediary position of the water column in stratified waters, but also throughout the euphotic zone up to the surface at latitudes above 40°N/30°S | [8, 9]  NCMA web | |
| *Prochlorococcus marinus* MED4 | CCMP2389, CCMP1378, CCMP1986 | 43°12'N, 06°52'E  Mediterranean Sea 5m depth | BX548174 (G) | Clust HLI; | Elongated, non-motile,  L: 1.2-1.6 µm  high light adapted  No PBS | T_isol_: ND  T_min_: 17°C  T_max_: 27°C  T_opt_: ND  T_main_: 20°C | Present in less stratified surface waters, predominantly between 35° and 48°N and 35° and 40°S | [8–10]  NCMA web | |
| *Prochlorococcus* *marinus* MIT9301 | CCMP2971 | 34.1667° N 66.3° W at Sargasso Sea depth 90m | CP000576 (G) |  | Elongated, non-motile,  L: 1.2-1.6 µm  low light adapted  No PBS | T_isol_: ND  T_min_: 18°C  T_max_: 22°C  T_opt_: ND  T_main_: 20°C |  | [11, 12]  NCMA web | |
| *Prochlorococcus* sp. MIT9215 | CCMP2939 | Equatorial Pacific, surface water | AF115271 (R) | Clust HLII; | Elongated, non-motile,  L: 1.2-1.6 µm  No PBS | T_isol_: ND  T_min_: 18°C  T_max_: 22°C  T_opt_: ND  T_main_: 20°C |  | [11, 12]  NCMA web | |
| *Prochlorococcus* sp. MIT9312 | CCMP2777 | 37.5002° N 68.2334° W at North Atlantic, Gulf Stream, depth 135m | AF053398 (R) | Clust HLII | Elongated, non-motile, L: 1.2-1.6 µm  No PBS | T_isol_: ND  T_min_: 19°C  T_max_: 26°C  T_opt_: ND  T_main_: 24°C |  | [8, 11, 12]  NCMA web | |
| *Synechococcus* sp. RCC307 | MinSyn016 | 39.17N 6.17E  Mediterranean Sea, depth 15m | NC_009482 (G) | MarClust_5.3 | Coccoid, non-motile, L: 1.0 µm  Pigment: T3b | T_isol_: <13°C  T_min_: 19°C  T_max_: 26°C  T_opt_: ND  T_main_: 20°C |  | [4, 5, 10]  RCC web | |
| *Synechococcus* sp. WH8016 | RCC2535 | 41.52N 70.67W  Woods Hole | AY172834 (R) | Mar.Clust_5.1A_I | Coccoid, non-motile, L: 1.0 µm  Pigment: T3a | T_isol_: 16-18.9 °C  T_min_: ND  T_max_: ND  T_opt_: ND  T_main_: 22°C |  | [6, 11, 13]  RCC Web | |
| *Synechococcus* sp. WH 8020 | RCC2437 | 38.68 N 69.32W Sargasso sea, depth 50m | CP011941 (G) | Mar.Clust_5.1A_I | Coccoid, non-motile, L: 1.0 µm  Pigment: T3dCA | T_isol_: 16-18.9 °C  T_min_: ND  T_max_: ND  T_opt_: ND  T_main_: 22°C |  | [4, 6, 14]  RCC Web | |
| *Synechococcus* sp. CC9311 | RCC1086 | 32.00N, 124.30W  Pacific Ocean, California current, coastal region, depth 95m | NC_008319 (G) | Mar.Clust_5.1A_I | Elongated, non-motile  L: 1.0 µm  Pigment: T3dCA | T_isol_: 16-18.9 °C  T_min_: 15 °C  T_max_: ND  T_opt_: ND  T_main_: 22°C |  | [4, 6, 8, 10, 12, 15, 16]  RCC web | |
| *Synechococcus* sp. RS9915 | RCC2553 | 29.47N 34.92E Red Sea, Gulf of Aqaba, Sampling depth 10 m | AY172825 (R) | Mar.Clust_5.1A_III | Coccoid, non-motile  L: 1.0 µm  Pigment: T3a | T_isol_: 25-27.9 °C  T_min_: ND  T_max_: ND  T_opt_: ND  T_main_: 20°C |  | [6]  RCC web | |
| *Synechococcus* sp. WH 8102 | RCC539  CCMP2370 | 22.48N 65.60W Atlantic Ocean, Sargasso Sea, pelagic water | NC_005070 (G) | Mar.Clust_5.1A_III | Coccoid, motile  L: 1.0 µm  Pigment: T3c | T_isol_: 25-27.9 °C  T_min_: 12 °C  T_max_: 30 °C  T_opt_: ND  T_main_: 20-24°C |  | [4, 6, 9, 10]  RCC Web  NCMA Web | |
| *Synechococcus* sp. WH 8103 | RCC29 | 28.50N 67.38W  Atlantic Ocean, pelagic surface water | NZ_LN847356 (D) | Mar.Clust_5.1A_III | Coccoid, non-motile  L: 1.0 µm  Pigment: T3c | T_isol_: 25-27.9 °C  T_min_: 12 °C  T_max_: 30 °C  T_opt_: ND  T_main_: 22°C |  | [3, 4, 6, 17] RCC Web | |
| *Synechococcus* sp. CC9902 | CCMP3074, RCC2673 | 32.15N 117.42W  Pacific Ocean, California current, coastal water Sampling depth 5m | CP000097 (G) | Mar_Clust_5.1A_IV | Coccoid, non-motile  L: 1.0 µm  Pigment: T3dCA | T_isol_: 13-15.9 °C  T_min_: ND  T_max_: 25°C  T_opt_: 18-22 °C  T_main_: 20°C | Typically a coastal specie | [4, 16, 18]  RCC web  NCMA Web | |
| *Synechococcus* sp. RS9904 | RCC543 | 29.47N 34.92E Red Sea, Gulf of Aqaba, pelagic water Sampling depth 10 m | AY172814 (R) | Mar.Clust_5.1A_II; | Coccoid, non-motile  L: 1.0 µm  Pigment: T3c | T_isol_: 25-27.9 °C  T_min_: ND  T_max_: ND  T_opt_: ND  T_main_: 22°C |  | [5]  RCC web | |
| *Synechococcus* sp. RS9908 | RCC547 | 29.47N 34.92E Red Sea, Gulf of Aqaba, pelagic water Sampling depth 10 m | AY172818 (R) | Mar.Clust_5.1A_II | Coccoid, non-motile  L: 1.0 µm  Pigment: T3a | T_isol_: >27.9 °C  T_min_: ND  T_max_: ND  T_opt_: ND  T_main_: 22°C |  | [5]  RCC web | |
| *Synechococcus* sp. RS9902 | RCC2376 | 29.47N 34.92E Red Sea, Gulf of Aqaba, pelagic water Sampling depth 1 m | AY172812 (R) | Mar.Clust_5.1A_II | Coccoid, non-motile  L: 1.0 µm  Pigment: T3c | T_isol_: 19—22.9 °C  T_min_: ND  T_max_: ND  T_opt_: ND  T_main_: 22°C |  | [5]  RCC web | |
| *Synechococcus* sp. CC9605 | CCMP3075, RCC753 | 30.42N, 123.97 W  Pacific Ocean California current, depth 51m | NC_007516 (G) | Mar.Clust_5.1A_II; | Elongated, motile  L: 1.0 µm  Pigment: T3c | T_isol_: 16—18.9 °C  T_min_: ND  T_max_: ND  T_opt_: ND  T_main_: 22-24°C | Typically found in surface oligotrophic waters | [4, 5, 8]  RCC web  NCMA web | |
| *Synechococcus* sp. WH 8109 | RCC2033 | 39.48N 70.47 W  Sargasso sea | NZ_CP006882 (G) | MarClust_5.1A_II | Coccoid, non-motile  L: 1.0 µm  Pigment: T3d | T_isol_: 19—21.9 °C  T_min_: ND  T_max_: ND  T_opt_: ND  T_main_: 22°C |  | [5]  RCC web | |
| *Synechococcus* sp. RS9916 | RCC555 | 29.47N 34.92E Red Sea, Gulf of Aqaba, Sampling depth 10 m | AY172826 (R) | Mar.Clust_5.1B_IX | Coccoid, non-motile  L: 1.0 µm  Pigment: T3c  T3dCA | T_isol_: 25—27.9 °C  T_min_: ND  T_max_: ND  T_opt_: ND  T_main_: 20-22°C |  | [4, 5, 10]  RCC web | |
| *Synechococcus* sp. WH8101 | RCC2555 | 41.52N 70.67W  Woods Hole  Pelagic surface water | AF001480 (R) | Mar.Clust_5.1B_VIII | Coccoid, non-motile  L: 1.0 µm  Pigment: T1 | T_isol_: <13 °C  T_min_: ND  T_max_: ND  T_opt_: ND  T_main_: 22°C |  | [3, 5]  RCC web | |
| *Synechococcus* sp. RS9914 | RCC553 | 29.47N 34.92E Red Sea, Gulf of Aqaba, pelagic water Sampling depth 10 m | AY172824 (R) | Mar.Clust_5.1B_VIII | Coccoid, non-motile  Pigment: T1 | T_isol_: 25—27.9 °C  T_min_: ND  T_max_: ND  T_opt_: ND  T_main_: 20 °C |  | [5]  RCC web | |
| *Synechococcus* sp. RS9917 | RCC556 | 29.47N 34.92E Red Sea, Gulf of Aqaba, pelagic water Sampling depth 10 m | NZ_CH724158 (D) | Mar.Clust_5.1B_VIII | Coccoid, non-motile  L: 1.0 µm  Pigment: T1 | T_isol_: 19—21.9 °C  T_min_: ND  T_max_: ND  T_opt_: ND  T_main_: 22°C |  | [4, 5, 10]  RCC web | |
| *Synechococcus* sp. MW73B4 |  | 47.58N, 13.66E  Lake Hallstatt | AY151250 (R) |  | Coccoid  Pigment: T1 | T_isol_: 15 °C  T_min_: ND  T_max_: ND  T_opt_: ND  T_main_: 15 °C | Low light 10 µmol photons m^-2^ s^-1^ | [19, 20] | |
| *Synechococcus* sp. BO8807 |  | 47.58N, 9.47E  Lake Constance, littoral zone | AF317074 (R) | Subalpine cluster I | Rods  L: 4.6 µm  Pigment: T2 | T_isol_: ND  T_min_: ND  T_max_: ND  T_opt_: ND  T_main_: 23 °C |  | [21, 22] | |
| *Synechococcus (Cyanobium)* sp. PCC7001 | *Synechococcus* sp. ATCC 27194  *Anacystis marina* NIBB 1098 | 40.85N 73.79W  City Island, New York, USA  Euryhaline strain isolated from soil and intertidal mud | AM709626 (R) |  | Oval, non-motile  L: 1.0 µm  Pigment: T1 | T_isol_: 19—21.9 °C  T_min_: ND  T_max_: 41 °C  T_opt_: 28 °C  T_main_: 22 °C |  | [3, 23]  PCC web | |
| *Synechococcus* sp. CCY9202 | BS5 | 55.14N, 14.92E  Baltic Sea, Bornholm  10 m depth,  salinity 9 gL^-1^ | AF330253 (R) |  | Pigment: T2 | T_isol_: ND  T_min_: ND  T_max_: ND  T_opt_: ND  T_main_: ND |  | [24, 25] | |
| *Synechococcus* sp. MW101C3 |  | 47.49N 13.23E  Lake Mondsee, Austria  Surface to 20m water column | AY151249 (R) |  | Oval  L: 1.0 µm  Pigment: T1 | T_isol_: 15 °C  T_min_: ND  T_max_: ND  T_opt_: 20 °C  T_main_: 15 °C | Low light 10 µmol photons m^-2^ s^-1^ | [19, 26–28] | |
| *Synechococcus* sp. BO0014 |  | 47.58N, 9.47E  Lake Constance, littoral zone | AF330251 (R) | Subalpine cluster II / Marine cluster 5.2 | Coccoid  Pigment: T2 | T_isol_: 21 °C  T_min_: ND  T_max_: ND  T_opt_: ND  T_main_: 21 °C |  | | [21, 22] |
| *Synechococcus* sp. BO8805 |  | 47.58N, 9.47E  Lake Constance, pelagic zone | AF317073 (R) | Subalpine cluster II / Marine cluster 5.2 | Coccoid  L: 1.4 µm  Pigment: T1 | T_isol_: 21 °C  T_min_: ND  T_max_: ND  T_opt_: ND  T_main_: 21 °C |  | | [20–22] |
| *Synechococcus* sp. WH5701 (RCC1084) | *Synechococcus bacillaris* | 41.10N, 72.88E  Atlantic Ocean Long Island Sound  Costal environment, euryhaline | CH724159 (D) | subalpine cluster II / Marine cluster 5.2 | Coccoid, non-motile  L: 1.0 µm  Pigment: T1 | T_isol_: 19—21.9 °C  T_min_: ND  T_max_: ND  T_opt_: ND  T_main_: 22 °C | Halotolerant but not marine  Considered as a transition strain between ɑ and β cyanobacteria | [4, 5, 7, 29–32]  RCC Web | |
| Synechococcus sp. BO8801 |  | 47.58N, 9.47E  Lake Constance | AF317071 (R) |  | Coccoid  L: 1.3 µm  Pigment: T1 | T_isol_: ND  T_min_: ND  T_max_: ND  T_opt_: ND  T_main_: 23 °C |  | [20] | |
| *Synechococcus* P211 |  | 73.16 N  78.30 W  Bylot Island, Canadian High Arctic  Tundra pond | AF098373 (R) |  | Coccoid, non-motile  L: 1.0 µm  Pigment: T1 | T_isol_: 13 °C  T_min_: ND  T_max_: ND  T_opt_: ND  T_main_: 13 °C | Optimal light intensity: 60 µmol photons m^-2^ s^-1^ | [2, 33] | |
| *Synechococcus* PS845 |  | Unknown coastal region of Russia | AF448070 (R) |  |  | T_isol_: ND  T_min_: ND  T_max_: ND  T_opt_: ND  T_main_: ND |  | [19] | |
| *Synechococcus elongatus* PCC7942 | *Synechococcus* RCC64, CALU 895; UTCC 100;  *Anacystis nidulans* NIBB 1113; *Synechococcus leopoliensis* UTEX 2434; IAM M-201; IAM M-204 ATCC 33912 | California USA  Stenohaline (freshwater) | NC_007604 (G) |  | Elongated, non-motile  L: 1.0 µm  Pigment: T1 | T_isol_: ND  T_min_: 15 °C  T_max_: 47.5 °C  T_opt_: 37 °C  T_main_: 22 °C | Model cyanobacterium | [34, 35]  RCC Web  PCC web | |
| *Synechococcus* sp. PCC73109 | *Agmenellum quadruplicatum* NIBB 1099, ATCC29404 | 40.85N 73.79W City Island, New York, USA  Sea water isolate | AB015061 (R) | Mar.Clust C ClassI; | Elongated, non-motile  L: 1.5 µm  Pigment: T1 | T_isol_: ND  T_min_: ND  T_max_: ND  T_opt_: 28 °C  T_main_: 22 °C |  | [3, 17]  PCC web | |
| *Synechococcus* sp. PCC7117 |  | Low salinity brine pond Port Hedland, Western Australia  Euryhaline | AB015060 (R) | Mar.Clust C ClassI | Elongated, non-motile  L: 1.5 µm  Pigment: T1 | T_isol_: ND  T_min_: ND  T_max_: ND  T_opt_: 28 °C  T_main_: 22 °C |  | [3, 17]  PCC web | |
| *Synechococcus* sp. PCC7002 | *Agmenellum quadruplicatum* ACMM 326; NIBB 1035, NIBB 1111; CCAP 1400/1 | 17.97N 67.04W  Magueyes Island, Puerto Rico  Fish pens, mud  Euryhaline estuarine | NC_010475 (G) | Mar.Clust C ClassI | Elongated, non-motile  L: 1.5 µm  Pigment: T1 | T_isol_: ND  T_min_: 15 °C  T_max_: 43 °C  T_opt_: 38 °C  T_main_: 22 °C | Fast growing strain doubling times < 4 h  Tolerates very high light up to 5000 µE m^-2^ s^-1^ | [9, 23, 36–38]  PCC web | |
| *Synechococcus* sp. PCC7003 | *Synechococcus* ACMM 327, ATCC 27265 *Coccochloris elabens* | 41.04 N, 73.61W  Greenwich, Connecticut, USA  Soil, sand sample at edge of clam bed  marine isolate | AB015059 (R) | Mar.Clust C ClassI | Oval, non-motile  L: 2.0 µm  Pigment: T1 | T_isol_: ND  T_min_: 15 °C  T_max_: 39 °C  T_opt_: 28 °C  T_main_: 22 °C |  | [3, 23]  PCC web | |
| *Gloeobacter violaceus* PCC7421 | *Gloeobacter* violaceus ACMM 428, DCC D0637, SAG 7.82  *Gloeothece coerulea* | 47.10N 8.19E  Lake Lucerne, Horn, Switzerland isolated from Rock, calcareous  stenohaline | NC_005125 (G) |  | Oval, non-motile  L: 1.0 µm  Pigment: T2 | T_isol_: ND  T_min_: 15 °C  T_max_: 39 °C  T_opt_: 28 °C  T_main_: 22 °C | *Gloeobacter violaceus* is a primordial cyanobacterium, the deepest- branching line of descent among all the cyanobacteria | [39]  PCC web | |

*G-whole genome; D-draft genome; R-16S rRNA gene sequence

**References:**

1. Powell LM, Bowman JP, Skerratt JH, Franzmann PD, Burton HR. Ecology of a novel Synechococcus clade occurring in dense populations in saline Antarctic lakes. Mar Ecol Prog Ser. 2005;291:65–80.

2. Vincent WF, Bowman JP, Rankin LM, Mcmeekin TA. Phylogenetic diversity of picocyanobacteria in Arctic and Antarctic ecosystems. In: Bell C, Brylinsky M, Johnson-Green M, editors. Microbial biosystems: new frontiers. Proceedings of the 8th international symposium on microbial ecology. Atlantic Canada Society for Microbial Ecology, Halifax, Canada; 2000. p. 317–22.

3. Honda D, Yokota A, Sugiyama J. Detection of seven major evolutionary lineages in cyanobacteria based on the 16S rRNA gene sequence analysis with new sequences of five marine Synechococcus strains. J Mol Evol. 1999;48:723–39.

4. Six C, Thomas JC, Garczarek L, Ostrowski M, Dufresne A, Blot N, et al. Diversity and evolution of phycobilisomes in marine Synechococcus spp.: A comparative genomics study. Genome Biol. 2007;8.

5. Pittera J, Humily F, Thorel M, Grulois D, Garczarek L, Six C. Connecting thermal physiology and latitudinal niche partitioning in marine Synechococcus. ISME J. 2014;8:1221–36.

6. Pittera J, Partensky F, Six C. Adaptive thermostability of light-harvesting complexes in marine picocyanobacteria. ISME J. 2017;11:1–13.

7. Pittera J, Jouhet J, Breton S, Garczarek L, Partensky F, Maréchal É, et al. Thermoacclimation and genome adaptation of the membrane lipidome in marine Synechococcus. Environ Microbiol. 2018;20:612–31.

8. Xiaoyuan C, Yang Q, Zhao F, Qin S, Yang Y, Shen J, et al. Comparative analysis of fatty acid desaturases in cyanobacterial genomes. Comp Funct Genomics. 2008;:1–26.

9. Scanlan DJ, Ostrowski M, Mazard S, Dufresne A, Garczarek L, Hess WR, et al. Ecological Genomics of Marine Picocyanobacteria. Microbiol Mol Biol Rev. 2009;73:249–99. doi:10.1128/MMBR.00035-08.

10. Dufresne A, Ostrowski M, Scanlan DJ, Garczarek L, Mazard S, Palenik BP, et al. Unraveling the genomic mosaic of a ubiquitous genus of marine cyanobacteria. Genome Biol. 2008;9:1–16.

11. Rocap G, Distel DL, Waterbury JB, Chisholm SW. Resolution of Prochlorococcus and Synechococcus Ecotypes by Using 16S-23S Ribosomal DNA Internal Transcribed Spacer Sequences. Appl Environ Microbiol. 2002;68:1180–91.

12. Zhaxybayeva O, Doolittle WF, Papke RT, Gogarten JP. Intertwined Evolutionary Histories of Marine Synechococcus and Prochlorococcus marinus. Genome Biol Evol. 2009;1:325–39. doi:10.1093/gbe/evp032.

13. Coutinho F, Tschoeke DA, Thompson F, Thompson C. Comparative genomics of Synechococcus and proposal of the new genus Parasynechococcus. PeerJ. 2016;4:e1522:1–18.

14. Everroad C, Six C, Partensky F, Thomas J-C, Holtzendorff J, Michelle A, et al. Biochemical Bases of Type IV Chromatic Adaptation in Marine Synechococcus spp. J Bacteriol. 2006;188:3345–56.

15. Cai H, Wang K, Huang S, Jiao N, Chen F. Distinct patterns of picocyanobacterial communities in winter and summer in the Chesapeake Bay. Appl Environ Microbiol. 2010;76:2955–60.

16. Apple JK, Strom SL, Palenik B, Brahamsha B. Variability in Protist Grazing and Growth on Different Marine Synechococcus Isolates ᰔ. Appl Environ Microbiol. 2011;77:3074–84.

17. Robertson BR, Watanabe MM. Phylogenetic analyses of Synechococcus strains ( cyanobacteria ) using sequences of 16S rDNA and part of the phycocyanin operon reveal multiple evolutionary lines and reflect phycobilin content. Int J Syst Evol Microbiol. 2001;51:861–71.

18. Hamilton TJ, Paz-Yepes J, Morrison RA, Palenik B, Tresguerres M. Exposure to bloom-like concentrations of two marine Synechococcus cyanobacteria ( strains CC9311 and CC9902 ) differentially alters fish behaviour. Conserv Physiol. 2014;2:1–9.

19. Crosbie ND, Pöckl M, Weisse T, Po M. Dispersal and Phylogenetic Diversity of Nonmarine Picocyanobacteria , Inferred from 16S rRNA Gene and cpcBA -Intergenic Spacer Sequence Analyses. Appl Environ Microbiol. 2003;69:5716–21.

20. Ernst A, Sandmann G, Postius C, Brass S, Kenter U, Boger P. Cyanobacterial Picoplankton from Lake Constance * II . Classification of Isolates by Cell Morphology and Pigment Composition. Bot Acta. 1992;105:161–7.

21. Ernst A, Becker S, Wollenzien UIA, Postius C. Ecosystem-dependent adaptive radiations of picocyanobacteria inferred from 16S rRNA and ITS-1 sequence analysis. Microbiology. 2003;149:217–28.

22. Becker S, Kumar A, Postius C, Peter B, Ernst A. Genetic diversity and distribution of periphytic Synechococcus spp . in biofilms and picoplankton of Lake Constance. FEMS Microbiol Ecol. 2004;49:181–90.

23. Stanier RY, Kunisawa R, Mandel M, Cohen-Bazire G. Purification and properties of unicellular blue-green algae (order Chroococcales). Bacteriol Rev. 1971;35:171–205.

24. Stomp M, van Dijk MA, van Overzee HMJ, Wortel MT, Sigon CAM, Egas M, et al. The Timescale of Phenotypic Plasticity and Its Impact on Competition in Fluctuating Environments. Am Nat. 2008;172:E169–85. doi:10.1086/591680.

25. Simis SGH, Huot Y, Babin M, Seppälä J, Metsamaa L. Optimization of variable fluorescence measurements of phytoplankton communities with cyanobacteria. Photosynth Res. 2012;112:13–30.

26. Callieri C, Amalfitano S, Corno G, Bertoni R. Grazing-induced Synechococcus microcolony formation: Experimental insights from two freshwater phylotypes. FEMS Microbiol Ecol. 2016;92:1–10.

27. Callieri C, Amalfitano S, Corno G, Di Cesare A, Bertoni R, Eckert EM. The microbiome associated with two Synechococcus ribotypes at different levels of ecological interaction. J Phycol. 2017;53:1151–8.

28. Callieri C, Coci M, Corno G, Macek M, Modenutti B, Balseiro E, et al. Phylogenetic diversity of nonmarine picocyanobacteria. FEMS Microbiol Ecol. 2013;85:293–301.

29. Varin T, Lovejoy C, Jungblut AD, Vincent WF, Corbeil J. Metagenomic analysis of stress genes in microbial mat communities from Antarctica and the high Arctic. Appl Environ Microbiol. 2012;78:549–59.

30. Xu Y, Jiao N, Chen F. Novel psychrotolerant picocyanobacteria isolated from Chesapeake Bay in the winter. J Phycol. 2015;51:782–90.

31. Acosta Pomar MLC, Caruso G, Maugeri TL, Scarfò R, Zaccone R. Distribution of Synechococcus spp. determined by immunofluorescent assay. J Appl Microbiol. 1998;84:493–500.

32. Rae BD, Förster B, Badger MR, Price GD. The CO2-concentrating mechanism of Synechococcus WH5701 is composed of native and horizontally-acquired components. Photosynth Res. 2011;109:59–72.

33. Vezina S, Vincent WF. Arctic cyanobacteria and limnological properties of their environment : Bylot Island , Northwest Territories , Canada ( 73° N , 80°W ). Polar Biol. 1997;7:523–34.

34. Porankiewicz J, Clarke AK. Induction of the Heat Shock Protein ClpB Affects Cold Acclimation in the Cyanobacterium Synechococcus sp . Strain PCC 7942. J Bacteriol. 1997;179:5111–7.

35. Eriksson MJ, Clarke a K. The heat shock protein ClpB mediates the development of thermotolerance in the cyanobacterium Synechococcus sp. strain PCC 7942. J Bacteriol. 1996;178:4839–46.

36. Berla BM, Saha R, Immethun CM, Maranas CD, Moon TS, Pakrasi HB. Synthetic biology of cyanobacteria: Unique challenges and opportunities. Front Microbiol. 2013;4 AUG:1–14.

37. Ludwig M, Bryant DA. Acclimation of the global transcriptome of the cyanobacterium Synechococcus sp. strain PCC 7002 to nutrient limitations and different nitrogen sources. Front Microbiol. 2012;3 APR:1–15.

38. Sakamoto T, Bryant D a. Synergistic effect of high-light and low temperature on cell growth of the Delta12 fatty acid desaturase mutant in Synechococcus sp. PCC 7002. Photosynth Res. 2002;72:231–42. doi:10.1023/A:1019820813257.

39. Turner SS, Pryer KM, Miao VPW, Palmer JD. Investigating deep phylogenetic relationships among cyanobacteria and plastids by small subunit rRNA sequence analysis. J Eukaryot Microbiol. 1999;46:327–38. doi:10.1111/j.1550-7408.1999.tb04612.x.

RCC Web:

PCC Web:

NCMA Web:
