# Supplementary figures and images for: Complete Genome Sequence and Comparative Analysis of Synechococcus sp. CS-601 (SynAce01), a Cold-Adapted Cyanobacterium from an Oligotrophic Antarctic Habitat
Source: Int J Mol Sci. 2019 Jan 3;20(1):152. doi: 10.3390/ijms20010152 (PMC6337551; doi:10.3390/ijms20010152)

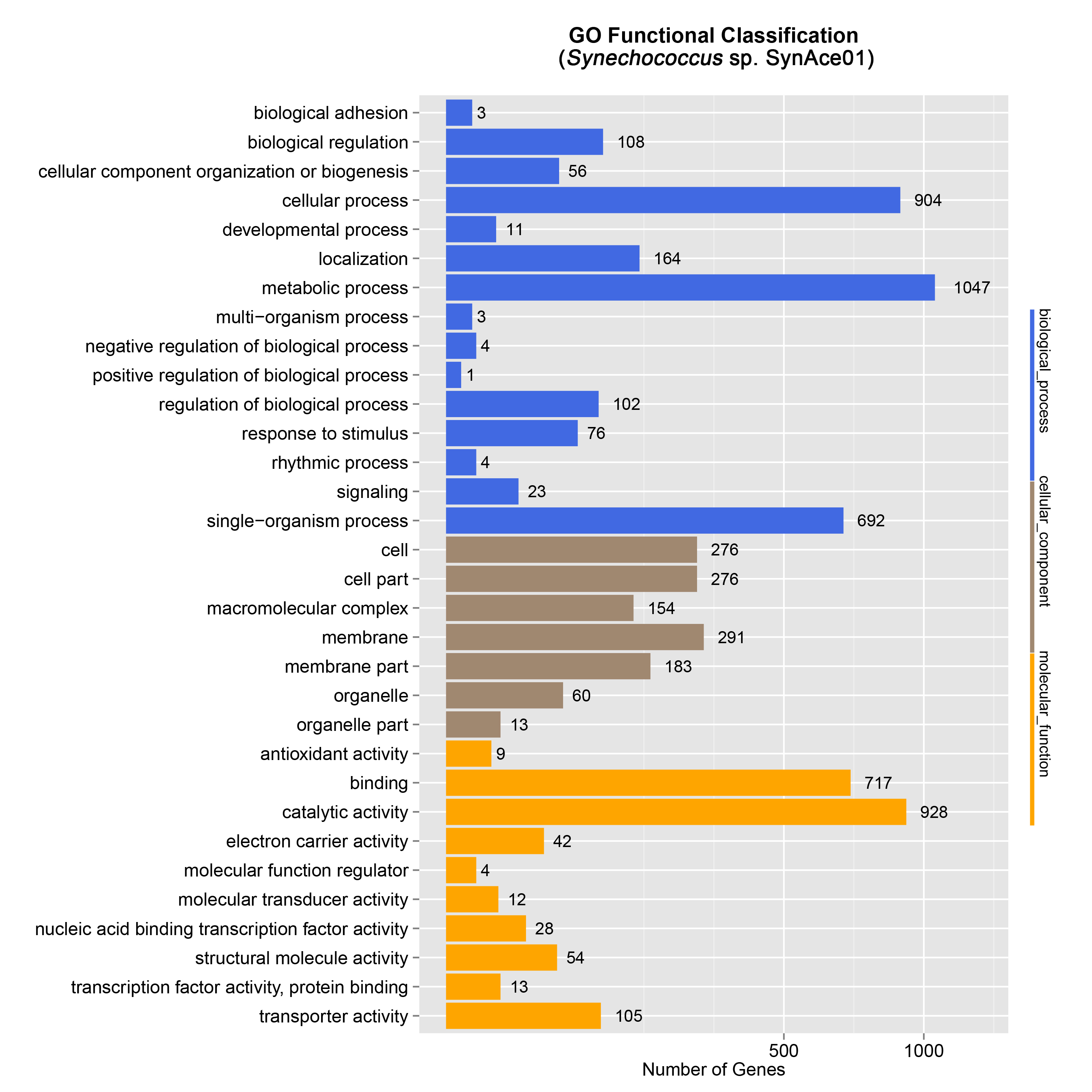

Supplement: Supplementary file 1 [file ijms-20-00152-s001.zip › Supplementary Figure S1.jpg]
