# Supplementary material for: Complete Genome Sequence and Comparative Analysis of Synechococcus sp. CS-601 (SynAce01), a Cold-Adapted Cyanobacterium from an Oligotrophic Antarctic Habitat
Source: Int J Mol Sci. 2019 Jan 3;20(1):152. doi: 10.3390/ijms20010152 (PMC6337551; doi:10.3390/ijms20010152)
Supplement: Supplementary file 1 [file ijms-20-00152-s001.zip › Supplementary Table S2.doc]

**Table S2 Prophage loci and genes within *Synechococcus* sp. SynAce01 genome**

| **Locus Tag** | **Start** | **End** | **Strand** | **Predicted Gene Product** | **Blast hit** |
| --- | --- | --- | --- | --- | --- |
| phiSynAce1 | 65524 | 73790 |  |  |  |
|  | 65524 | 67113 | - | hypothetical protein | PHAGE_Synech_S_CBS2_NC_015463: hypothetical protein; BM449_00310; phage (gi331027993) |
|  | 67217 | 67579 | + | prevent-host-death protein | prevent-host-death protein; BM449_00315 |
|  | 67543 | 68061 | + | DNA-binding protein | DNA-binding protein; BM449_00320 |
|  | 69446 | 70039 | - | type III RNAP sigma factor | PHAGE_Cyanop_PSS2_NC_013021: type III RNAP sigma factor; BM449_00325; phage (gi254729465) |
|  | 70046 | 71683 | - | hypothetical protein | PHAGE_Lactoc_98201_NC_031064: hypothetical protein; BM449_00330; phage (gi100031) |
|  | 71680 | 72201 | - | hypothetical protein | hypothetical protein; BM449_00335 |
|  | 72677 | 73060 | + | hypothetical protein | PHAGE_Agroba_7_7_1_NC_019519: hypothetical protein; BM449_00340; phage (gi435844376) |
|  | 73107 | 73790 | - | hypothetical protein | PHAGE_Clostr_phiCDHM11_NC_029001: hypothetical protein; BM449_00345; phage (gi971764697) |
| phiSynAce2 | 1186119 | 1204368 |  |  |  |
|  | 1186119 | 1186130 | + | *att*L | *att*L (ACAGGCCAGCGC) |
|  | 1192341 | 1192673 | + | HicB protein | PHAGE_Burkho_ST79_NC_021343: HicB protein; BM449_06095; phage (gi509141623) |
|  | 1192840 | 1193142 | + | hypothetical protein | PHAGE_Brucel_BiPBO1_NC_031264: hypothetical protein; BM449_06100; phage (gi100020) |
|  | 1193139 | 1193450 | + | hypothetical protein | PHAGE_Brucel_BiPBO1_NC_031264: hypothetical protein; BM449_06105; phage(gi100019) |
|  | 1194623 | 1194919 | + | hypothetical protein | hypothetical protein; BM449_06110 |
|  | 1195040 | 1195447 | + | hypothetical protein | hypothetical protein; BM449_06115 |
|  | 1195516 | 1196420 | - | integrase | PHAGE_Staphy_phiETA_NC_003288: similar to phage phi 11 integrase; BM449_06120; phage (gi17426229) |
|  | 1197381 | 1198334 | - | transposase | PROPHAGE_Xantho_33913: IS1595 transposase; BM449_06125; phage (gi21231554) |
|  | 1198325 | 1199302 | - | hypothetical protein | hypothetical protein; BM449_06130 |
|  | 1199344 | 1200639 | - | enolase | PHAGE_Strept_20617_NC_023503: enolase; BM449_06135; phage (gi588295080) |
|  | 1204368 | 1204379 | + | *att*R | *att*R (ACAGGCCAGCGC) |
